# Author Correction: GABA signalling modulates stomatal opening to enhance plant water use efficiency and drought resilience

**DOI:** 10.1038/s41467-024-46158-2

**Published:** 2024-02-26

**Authors:** Bo Xu, Yu Long, Xueying Feng, Xujun Zhu, Na Sai, Larissa Chirkova, Annette Betts, Johannes Herrmann, Everard J. Edwards, Mamoru Okamoto, Rainer Hedrich, Matthew Gilliham

**Affiliations:** 1 Plant Transport and Signalling Lab, ARC Centre of Excellence in Plant Energy Biology, Waite Research Institute, Glen Osmond, SA Australia; 2 School of Agriculture, Food and Wine, Waite Research Precinct, University of Adelaide, Glen Osmond, SA Australia; 3 College of Horticulture, Nanjing Agricultural University, Nanjing, China; 4 ARC Industrial Transformation Research Hub for Wheat in a Hot and Dry Climate, Waite Research Institute, University of Adelaide, Glen Osmond, SA Australia; 5 CSIRO Agriculture & Food, Glen Osmond, SA Australia; 6 Institute for Molecular Plant Physiology and Biophysics, University of Würzburg, Würzburg, Germany

**Keywords:** Plant transporters, Plant physiology, Plant signalling

Correction to: *Nature Communications* 10.1038/s41467-021-21694-3, published online 29 March 2021

The original version of this article contained an error in Fig. 3a, in which asterisks to display statistical significance were omitted above the box plots for WT 3d and WT 7d. In addition, the “Acknowledgements” section incorrectly referred to ARC Discovery grant DP21012828 and ARC Centre of Excellence CE14010008. The correct grant numbers are DP210102828 and CE140100008. These errors have now been corrected in both the PDF and HTML versions of the article.

