# Supplementary material for: GABA signalling modulates stomatal opening to enhance plant water use efficiency and drought resilience
Source: Nat Commun. 2021 Mar 29;12:1952. doi: 10.1038/s41467-021-21694-3 (PMC8007581; doi:10.1038/s41467-021-21694-3)
Supplement: Supplementary file 1 — Supplementary Information [file 41467_2021_21694_MOESM1_ESM.pdf]

GABA signalling modulates stomatal opening to enhance plant water use efficiency and drought resilience

Bo Xu[1,2], Yu Long[1,2], Xueying Feng[1,2], Xujun Zhu[1,3], Na Sai[1,2], Larissa Chirkova[2,4], Annette Betts[5], Johannes Herrmann[6], Everard J Edwards[5], Mamoru Okamoto[2,4], Rainer Hedrich[6] & Matthew Gilliham[1,2]*

[1] Plant Transport and Signalling Lab, ARC Centre of Excellence in Plant Energy Biology, Waite Research Institute, Glen Osmond, SA 5064, Australia

[2] School of Agriculture, Food and Wine, Waite Research Precinct, University of Adelaide, Glen Osmond, SA 5064, Australia

[3] College of Horticulture, Nanjing Agricultural University, Nanjing 210095, China

[4] ARC Industrial Transformation Research Hub for Wheat in a Hot and Dry Climate, Waite Research Institute, University of Adelaide, Glen Osmond, SA 5064, Australia

[5] CSIRO Agriculture & Food, Locked Bag 2, Glen Osmond, SA, 5064, Australia

[6] Institute for Molecular Plant Physiology and Biophysics, University of Würzburg, Würzburg 97070, Germany

SUPPLEMENTARY INFORMATION

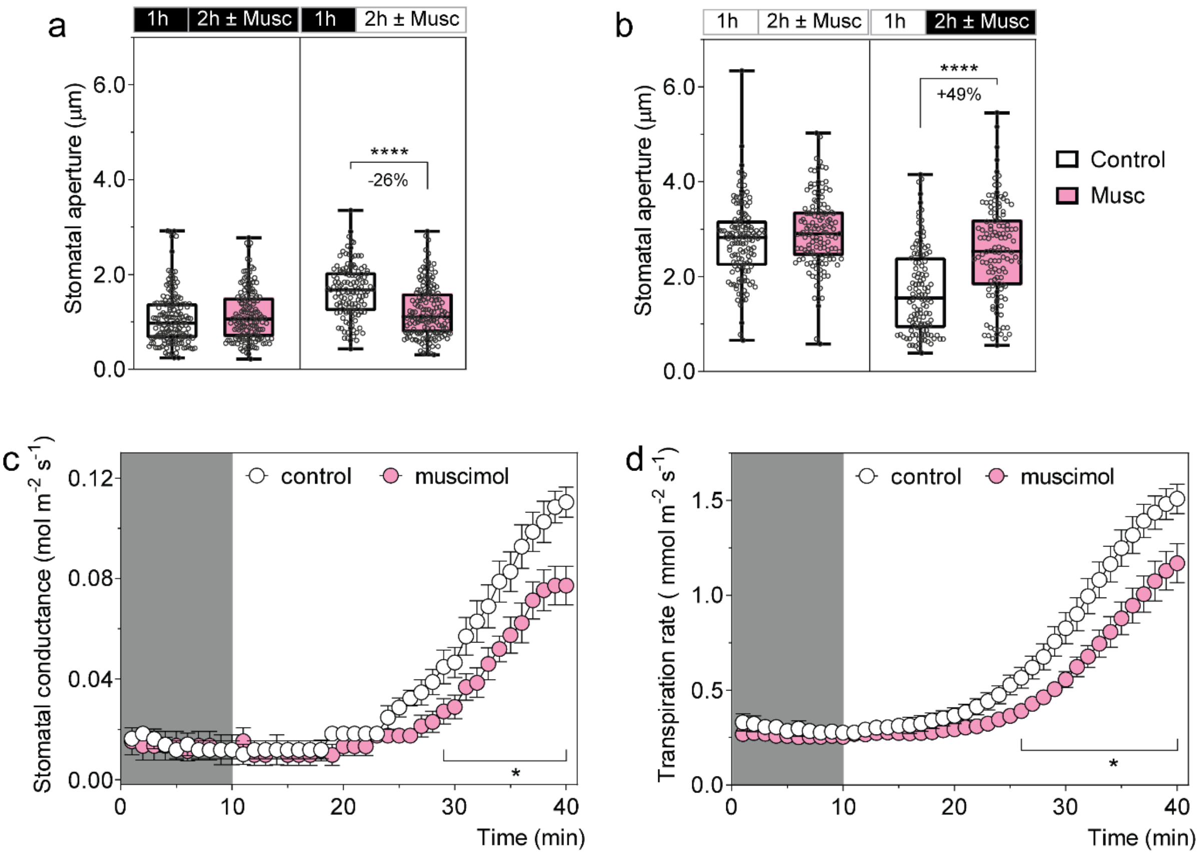

Supplementary Figure 1. Muscimol antagonises stomatal aperture changes initiated by light and dark treatments a-b, Exogenous muscimol application reduces stomatal pore movement in response to light or dark. Epidermal strips were pre-incubated in stomatal pore measurement buffer for 1 h under dark (**a**) or light (**b**), followed by 2 h incubation under constant dark (**a**), light (**b**), dark-to-light transition (**a**) or light-to-dark transition (**b**) as indicated above graphs by black (dark) or white (light) bars, together with the application of 10 µM muscimol (Musc); n = 156 for control (constant dark), n = 151 for muscimol (constant dark), n = 127 for control (dark-to-light transition) and n = 151 for muscimol (dark to light transition) (**a**); n = 134 for control (constant light), n = 132 for muscimol (constant light), n = 118 for control (light-to-dark transition) and n = 120 for muscimol (light-to-dark transition) (**b**). **c-d**, Muscimol feeding reduces stomatal conductance, transpiration rate of detached leaves. Stomatal conductance (**c**), transpiration rate (**d**) of detached leaves of wildtype Arabidopsis plants determined by LCpro-SD Portable Photosynthesis System was fed by artificial

xylem sap solution with or without 10 μM muscimol supplement; n = 7 for control and n = 8 for muscimol (**c, d**). All data are plotted with box and whiskers plots: whiskers plot represents minimum and maximum values, and box plot represents second quartile, median and third quartile (**a, b**) or data are represented mean ± s.e.m (**c, d**); statistical difference was determined by Two-way ANOVA (**a, b**), or two-sided Student's *t-test* (**c, d**), *P<0.05 and ****P<0.0001.

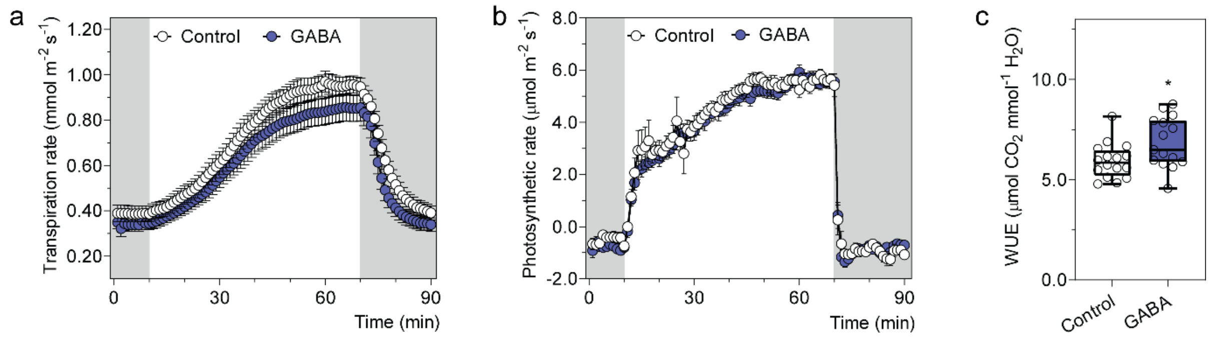

Supplementary Figure 2. GABA feeding increases WUE of detached leaves. GABA feeding reduces transpiration rate and increases water use efficiency (WUE) of detached leaves. Transpiration (**a**) and photosynthetic rate (**b**) of 5-6 week-old leaves of 5-6 week-old *A. thaliana* wildtype plants was recorded using a LiCor LI-6400XT in response to dark (shaded region) and 200 µmol m^{-2} s^{-1} light (white region), fed with artificial xylem sap solutions ± 4 mM GABA. **c**, WUE of detached leaves was calculated based on the ratio of photosynthetic rate (**b**) versus transpiration rate (**a**), n = 16 independent leaves for control and n = 15 for GABA (**a-c**). All data are plotted with box and whiskers plots: whiskers plot represents minimum and maximum values, and box plot represents second quartile, median and third quartile (**c**) or data are represented mean ± s.e.m (**a, b**), statistical difference was determined by two-sided Student's *t-test*, *P<0.05

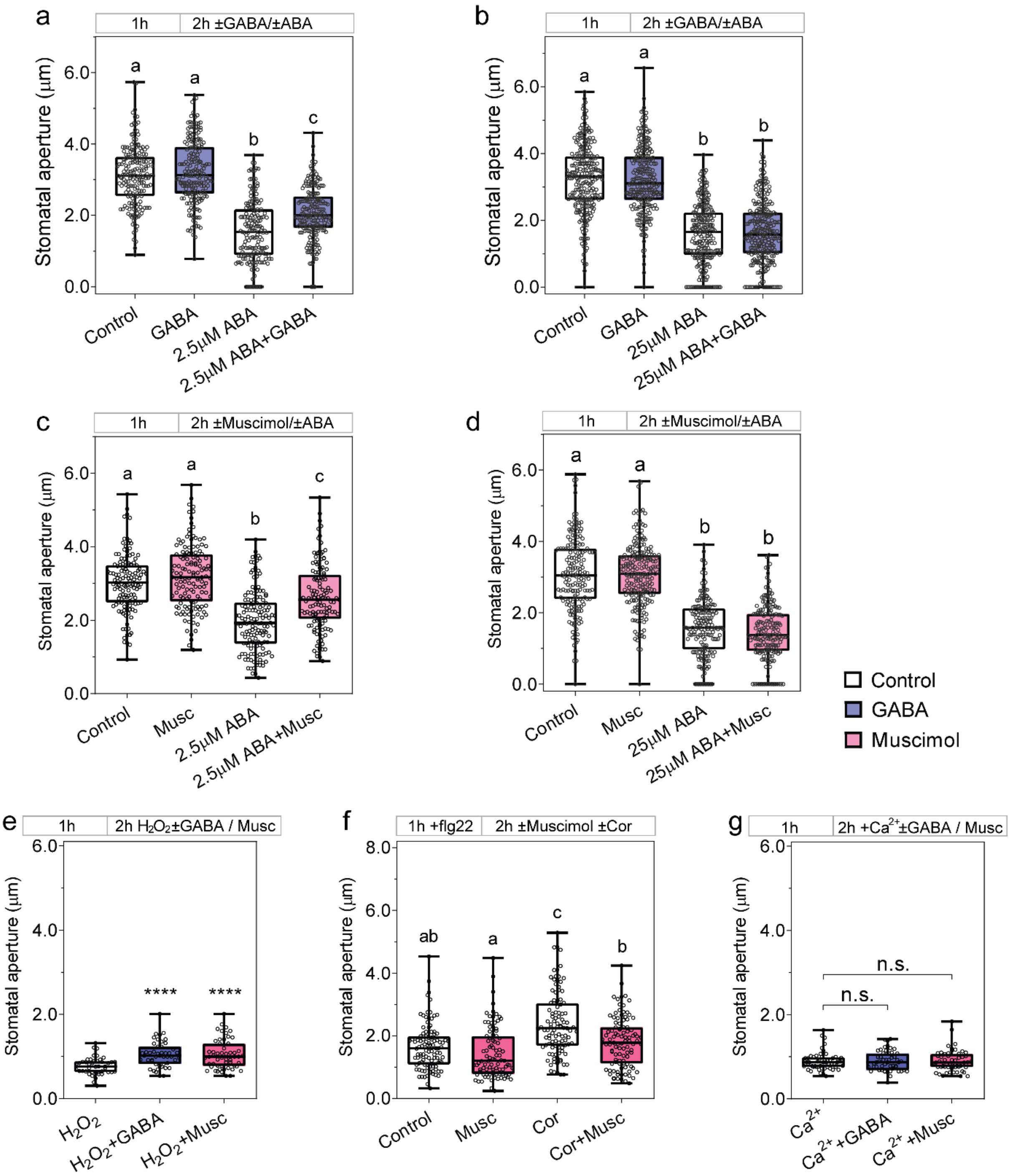

Supplementary Figure 3. GABA and muscimol inhibit stomatal aperture changes triggered by signaling molecules. a-g, Exogenous GABA or muscimol application reduces stomatal closure in response to 2.5 μM ABA (**a, c**), 50 μM H_2O_2[1] (**e**) and stomatal opening to 0.5 μg ml^{-1} coronatine[2] (**f**), but not to 25 μM ABA (**b, d**) and 2 mM $CaCl_2$[1] (**g**). Epidermal strips were pre-incubated in stomatal pore measurement buffer for 1 h under light, followed by 2 h treatment under light with or

without combination of ABA ± 2 mM GABA (**a, b**) / 10 μM muscimol (**c, d**), H_2O_2 ± 2 mM GABA/10 μM muscimol (Musc) (**e**), coronatine (Cor) ± 10 μM muscimol (**f**), and $CaCl_2$ (Ca^{2+}) ± 2 mM GABA/10 μM muscimol (**g**) as indicated; n = 183 for control, n = 191 for GABA, n = 171 for ABA and n = 201 for ABA+GABA (**a**); n = 249 for control, n = 249 for GABA, n = 243 for ABA and n = 261 for ABA+GABA (**b**); n = 133 for control, n = 137 for muscimol, n = 142 for ABA and n = 131 for ABA+muscimol (**c**); n = 180 for control, n = 222 for muscimol, n = 176 for ABA and n = 179 for ABA+muscimol (**d**); n = 44 for H_2O_2, n = 47 for H_2O_2+GABA and n = 54 for H_2O_2+Musc (**e**); n = 103 for control, n = 103 for Cor, n = 101 for Cor+muscimol and n = 91 for muscimol (**f**);); n = 52 for Ca^{2+}, n = 52 for Ca^{2+}+GABA and n = 54 for Ca^{2+}+Musc (**g**). All data are plotted with box and whiskers plots: whiskers plot represents minimum and maximum values, and box plot represents second quartile, median and third quartile; statistical difference as determined by One-Way ANOVA (**a-g**), **** P<0.0001, a, b and c represent groups without significant difference, P< 0.05 (**a-d, f**).

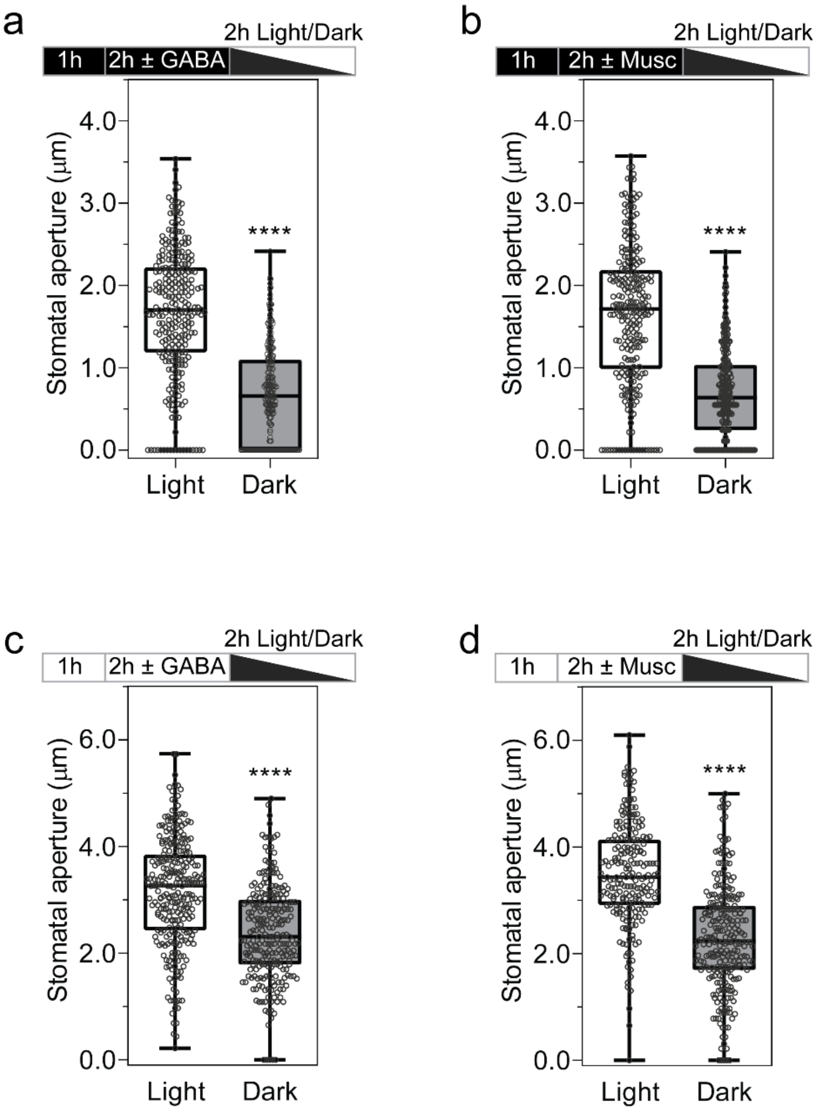

Supplementary Figure 4. Guard cells are viable after treatment with GABA and muscimol. a-d, Guard cells were competent in movement after removal of GABA or muscimol treatments, as closed pores opened when exposed to light (**a-b**) or open pores closed when exposed to dark (**c-d**) following removal of GABA or muscimol. Epidermal strips were incubated under dark (**a-b**) or light (**c-d**) for 1 h, followed by 2 h treatment of 2 mM GABA (**a, c**) or 10 μM muscimol (**b, d**), then epidermal strips were transferred into fresh stomatal measurement buffer with 2 h light or dark treatment before measurement; n = 272 for light and n = 247 for dark (**a**); n = 251 for light and n = 251 for dark (**b**); n = 275 for light and n = 248 for dark (**c**); n = 217 for light and n = 261 for dark (**d**). All data are plotted with box and whiskers plots:

whiskers plot represents minimum and maximum values, and box plot represents second quartile, median and third quartile; statistical difference as determined by two-sided Student's *t*-test (**a-d**), **** $P<0.0001$.

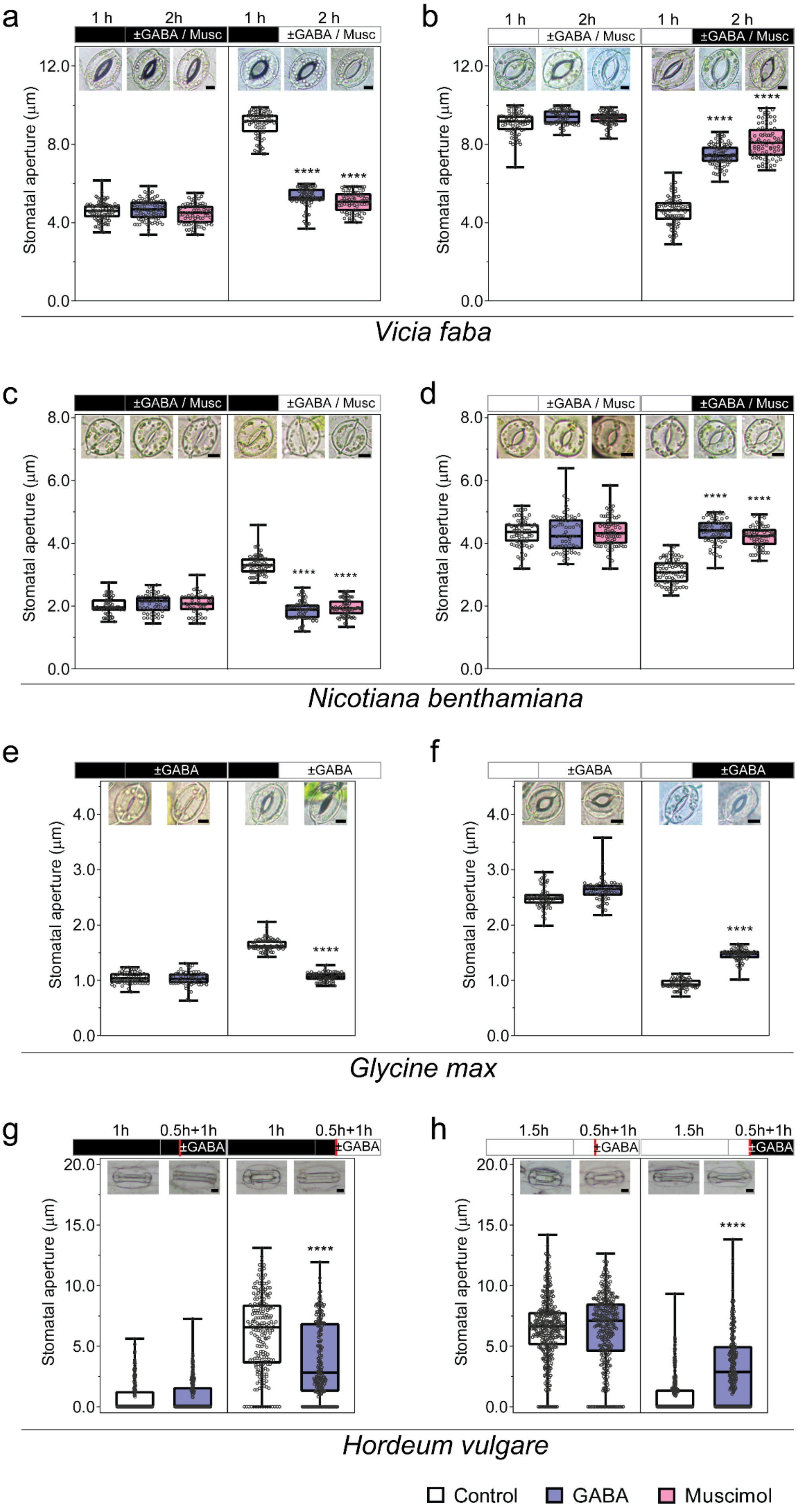

a
1 h
2h
±GABA / Musc
1 h
2 h
±GABA / Musc
Stomatal aperture (µm)
12.0
8.0
4.0
0.0
****
****
b
1 h
2h
±GABA / Musc
1 h
2 h
±GABA / Musc
****
****
Vicia faba
c
±GABA / Musc
±GABA / Musc
8.0
6.0
4.0
2.0
0.0
****
****
d
±GABA / Musc
±GABA / Musc
****
****
Nicotiana benthamiana
e
±GABA
±GABA
4.0
3.0
2.0
1.0
0.0
****
f
±GABA
±GABA
****
Glycine max
g
1h
0.5h+1h
±GABA
1h
0.5h+1h
±GABA
20.0
15.0
10.0
5.0
0.0
****
h
1.5h
0.5h+1h
±GABA
1.5h
0.5h+1h
±GABA
****
Hordeum vulgare
Control
GABA
Muscimol

Supplementary Figure 5. GABA and muscimol inhibit stomatal aperture changes in response to light and dark in *Vicia faba* (broad bean), *Nicotiana benthamiana* (tobacco), *Glycine max* (soybean) and *Hordeum vulgare* (barley). Epidermal strips were pre-incubated in stomatal measurement buffer for 1 h under dark (**a, c, e**) or light (**b, d, f**), followed by 2 h incubation under constant dark (**a, c, e**), light (**b, d, f**) as illustrated by black (dark) or white (light) bars, ± 2 mM GABA or 10 µM muscimol (Musc) as indicated; barley leaf samples were first detached and bathed in a modified measurement buffer under 2h dark (**g**) or light (100 µmol m^{-2} s^{-1}) (**h**), then pre-treated in the fresh buffer ±1 mM GABA for 0.5 h as indicated by black or white bars; after this pre-treatment (break by red line), leaf samples were incubated in continuous dark (**g**), light (**h**), dark-to-light (**g**) or light-to-dark (**h**) transition for additional 1 h before the epidermal strips were peeled for imaging. n = 88 for control (constant dark), n = 85 for GABA (constant dark), n = 82 for muscimol (constant dark), n = 78 for control (dark-to-light transition), n = 106 for GABA (dark-to-light transition) and n = 76 for muscimol (dark-to-light transition) (**a**); n = 73 for control (constant light), n = 65 for GABA (constant light), n = 76 for muscimol (constant light), n = 89 for control (light-to-dark transition), n = 85 for GABA (light-to-dark transition) and n = 84 for muscimol (light-to-dark transition) (**b**); n = 50 for control (constant dark), n = 52 for GABA (constant dark), n = 50 for muscimol (constant dark), n = 63 for control (dark-to-light transition), n = 65 for GABA (dark-to-light transition) and n = 64 for muscimol (dark to light transition) (**c**); n = 73 for control (constant light), n = 60 for GABA (constant light), n = 78 for muscimol (constant light), n = 78 for control (light-to-dark transition), n = 67 for GABA (light-to-dark transition) and n = 65 for muscimol (light-to-dark transition) (**d**); n = 61 for control (constant dark), n = 60 for GABA (constant dark), n = 63 for control (dark-to-light transition) and n = 62 for GABA (dark-to-light transition) (**e**); n = 59 for control (constant light), n = 59 for GABA (constant light), n = 60 for control (light-to- dark transition) and

n = 60 (dark-to-light transition) (**f**); n = 177 for control (constant dark), n = 220 for GABA (constant dark), n = 201 for control (dark-to-light transition) and n = 203 for GABA (dark-to-light transition) (**g**); n = 350 for control (constant light), n = 301 for GABA (constant light), n = 289 for control (light-to-dark transition) and n = 228 for GABA (light-to dark-transition) (**h**). All data are plotted with box and whiskers plots: whiskers plot represents minimum and maximum values, and box plot represents second quartile, median and third quartile; statistical difference was determined by Two-way ANOVA, ****$P < 0.0001$; scale bars = 5 μm (**a-h**).

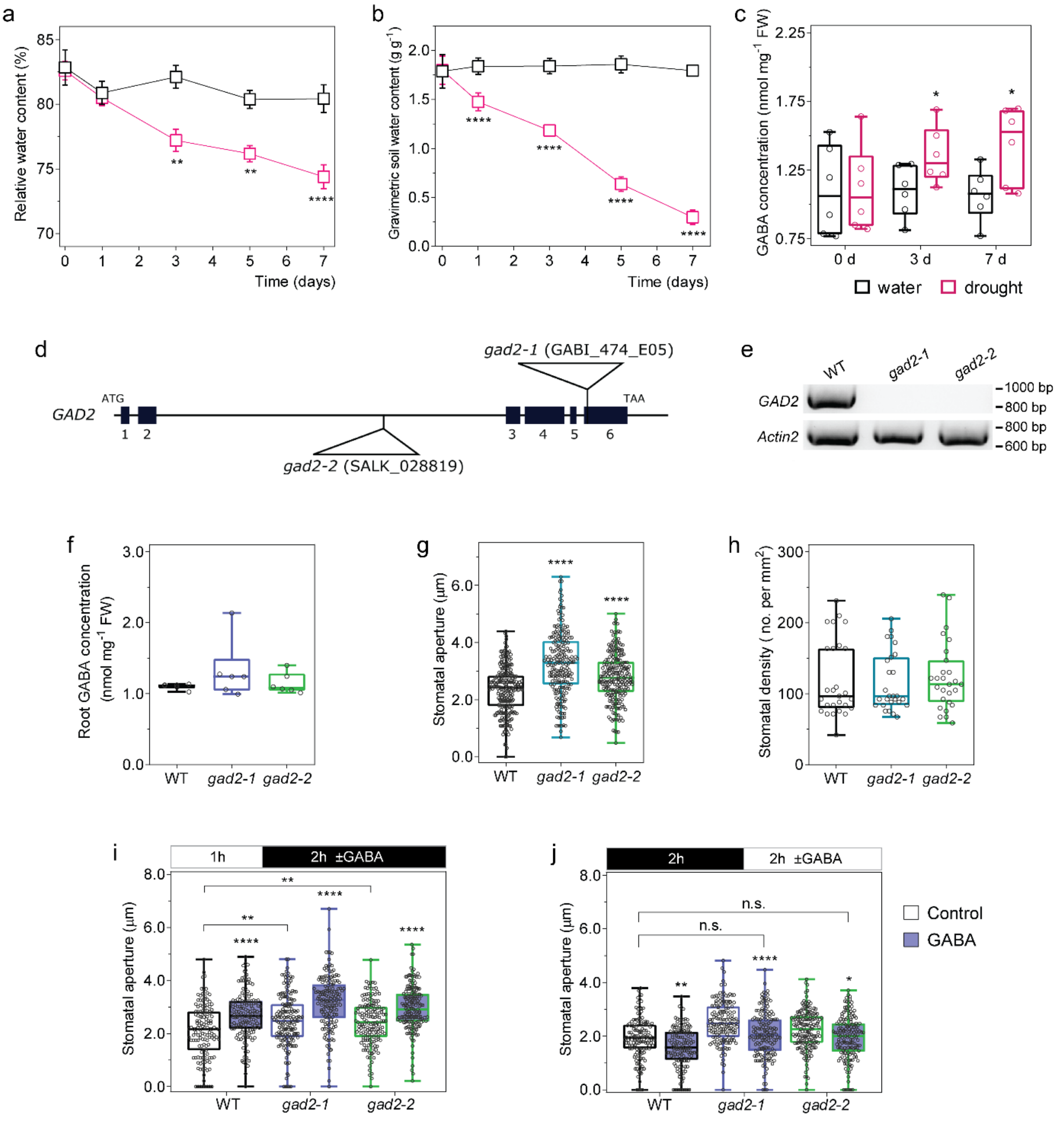

Supplementary Figure 6. GABA accumulates in leaves of Arabidopsis upon drought, and *gad2* knockout plants have greater stomatal apertures but show wildtype (WT)-like responses to exogenous GABA and root GABA accumulation. **a,** Relative water content in wildtype Arabidopsis leaves under well-water (black) or drought (red) treatments as indicated. **b,** Water content in the potted soil corresponding to the plants sampled in (**a**). **c,** Leaf GABA concentration of wildtype Arabidopsis following well-watered control treatment (black) or drought (red). 2-3 leaves per plant were sampled for relative water content measurement, as shown in

(**a**); the whole rosette from the sampled plant in (**a**) was harvested and snap frozen in liquid nitrogen for later GABA measurement, as shown in (**c**); and the pot soil of corresponding plants harvested in (**a, b**) was sampled to determine gravimetric soil water content, as shown in (**b**); n = 6 (**a-c**). **d,** A diagram of *GAD2* T-DNA insertional mutant alleles in the Arabidopsis genome. **e,** Reverse transcriptional PCR semi-quantification of *GAD2* transcripts in Arabidopsis WT and *gad2* knockout plants, *Actin2* used as an internal control, similar results were obtained from three independent biological replicates. **f,** Root GABA concentration of WT, *gad2-1* and *gad2-2* plants. Roots were harvested from 5-6 week-old plants grown hydroponically in basal nutrient solution (2 mM NH_4NO_3, 3 mM KNO_3, 0.1 mM $CaCl_2$, 2 mM KCl, 2 mM $Ca(NO_3)_2$, 2 mM $MgSO_4$, 0.6 mM KH_2PO_4, 1.5 mM NaCl, 50 μM NaFe(III)EDTA, 50 μM H_3BO_3, 5 μM $MnCl_2$, 10 μM $ZnSO_4$, 0.5 μM $CuSO_4$, 0.1 μM Na_2MoO_3, pH = 5.6 by KOH)[3], n = 6 plants. **g-j,** Stomatal aperture and density on the leaf abaxial side of Arabidopsis WT and *gad2* knockouts; epidermal strips were peeled and incubated in stomatal measurement buffer for 1 h under light before measurement n = 254 for WT, n = 215 for *gad2-1* and n = 226 for *gad2-2* (**g**); n = 27 sampling areas (0.57 x 0.42 mm) consisting of three areas per leaf, three leaves per plant and three plants per line sampled (**h**). **i-j,** Epidermal strips were pre-incubated in stomatal measurement buffer for 1 h under light (**i**) or 2 h dark (**j**), followed by 2 h incubation dark (**i**) or light (**j**) as indicated by black (dark) or white (light) bars ± blind treatment of 2 mM GABA or control; n = 135, 166 and 157 for WT, *gad2-1* and *gad2-2* with control treatment, n = 146, 162 and 174 for WT, *gad2-1* and *gad2-2* with GABA treatment (**i**); n = 139, 155 and 160 for WT, *gad2-1* and *gad2-2* with control treatment, n = 136, 155 and 153 for WT, *gad2-1* and *gad2-2* with GABA treatment (**j**). All data are plotted with box and whiskers plots: whiskers plot represents minimum and maximum values, and box plot represents second quartile, median and third quartile (**c, f-j**); or data are represented

by mean ± s.e.m. (**a, b**); statistical difference was determined by two-sided Student's *t*-test (**c**), One-way ANOVA (**f-h**) or Two-way ANOVA (**a, b, i, j**), $^{*}P < 0.05$, $^{**}P < 0.01$ and $^{****}P < 0.0001$.

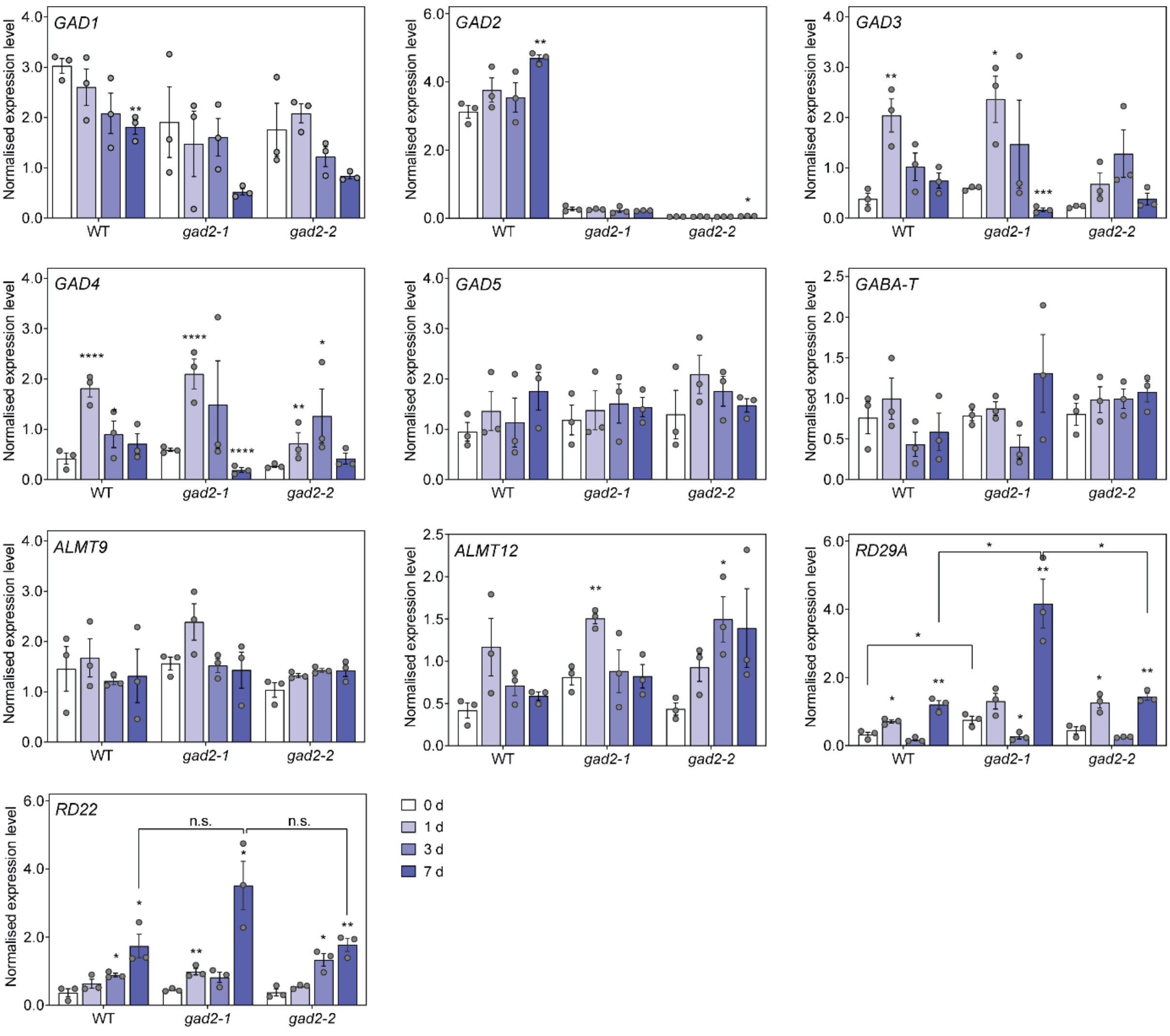

Supplementary Figure 7. ***gad2* knockouts have similar transcriptional profiles to wildtype plants of other *GADs*, *GABA-T*, *ALMT9*, *ALMT12* or ABA responsive genes under drought stress.** Quantitative real time PCR of *GAD1*, *GAD2*, *GAD3*, *GAD4*, *GAD5, GABA-T*, *ALMT9*, *ALMT12* and ABA marker genes –*RD29A* and *RD22*[4] in the leaves of Arabidopsis wildtype (WT), *gad2-1* and *gad2-2* plants following drought treatment for 0, 1, 3 and 7 days as indicated, expression levels were normalized against three control genes –*Actin2*, *EF1α* and *GAPDH-A*; data are represented by means ± s.e.m; n = 3, statistical difference as determined via the comparison of genes from drought-treated plants (1, 3 and 7 days) with non-droughted (0 day) plants within the same genotype by two-sided Student's *t-test*, **P< 0.05* ***P< 0.01*, ****P< 0.001* and *****P< 0.0001*.

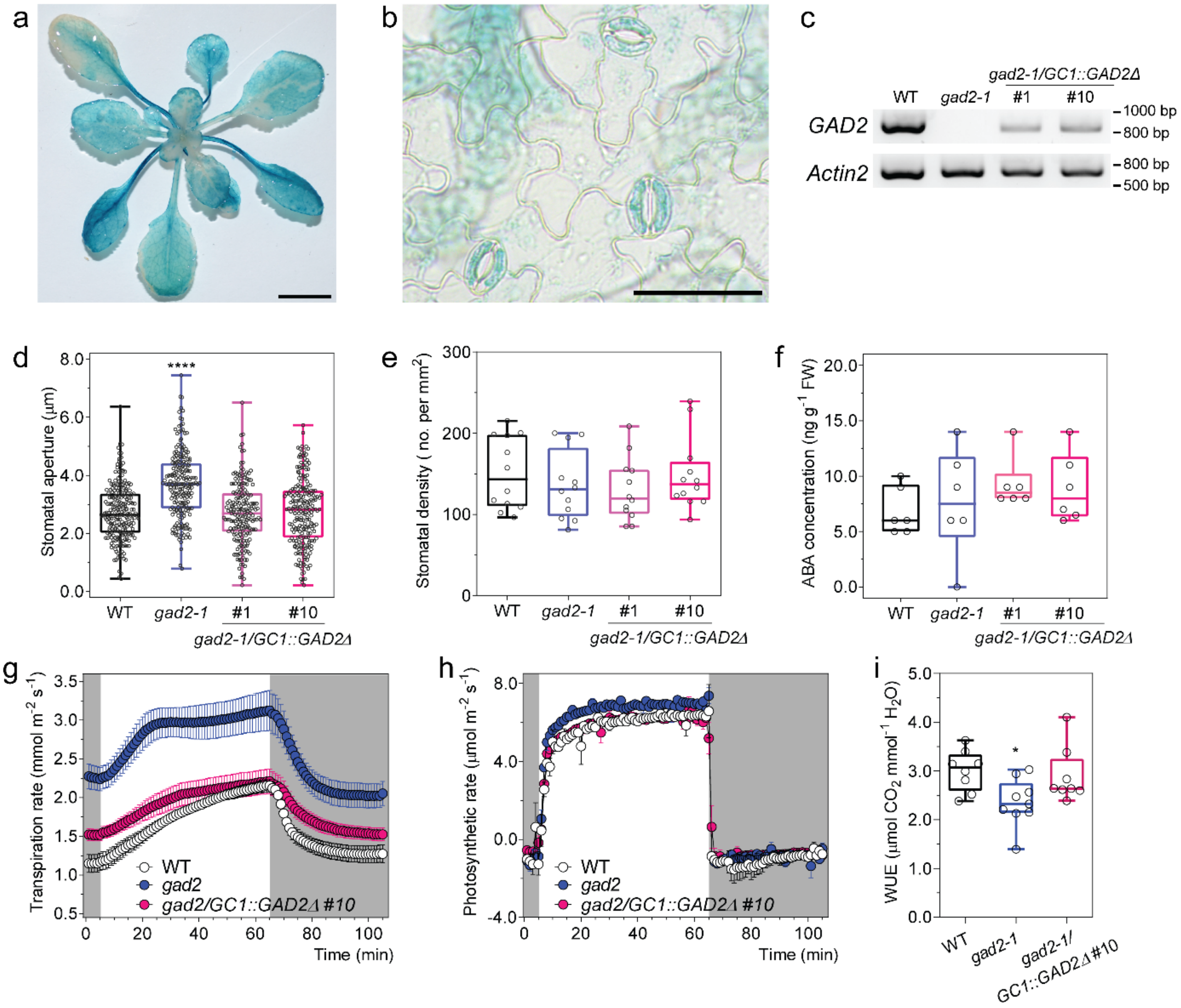

Supplementary Figure 8. *GAD2* is highly expressed in leaves and guard cells, and guard-cell cell complementation of *GAD2Δ* restores stomatal aperture and WUE without modifying stomatal density. a-b, Representative GUS histochemical staining of *pGAD2::GUS* whole rosette**;** image of 3-4 week-old *pGAD2::GUS* plants after staining in histochemical staining buffer, GUS staining of the guard cells was observed in all plants examined that were expressing *pGAD2::GUS*, scale bar = 5 mm (**a**) and epidermal peels from 3-4 week-old *pGAD2::GUS* leaves (**a**), scale bar = 50 µm (**b**). **c,** Reverse-transcriptional PCR quantification of *GAD2* transcripts in Arabidopsis wildtype (WT), *gad2-1*, *gad2-1/GC1::GAD2Δ* #1 and #10 plants, similar results were obtained from three independent biological replicates. **d-e,** Stomatal aperture (**d**) and density (**e**) on the leaf abaxial side of Arabidopsis WT, *gad2-1*, *gad2-*

1/GC1::GAD2Δ #1 and #10 plants; epidermal strips were peeled and incubated in stomatal measurement buffer for 1 h under light before measurement, n = 223 for WT, n = 212 for *gad2-1*, n = 197 for *gad2-1/GC1::GAD2Δ* #1 and n = 224 for *gad2-1/GC1::GAD2Δ* #10 (**d**); n = 12 leaf areas (0.57 x 0.42 mm); two areas per leaf, two leaves per plant and three plants per line were sampled (**e**). **f**, ABA accumulation in rosette leaves of 5-6 week-old Arabidopsis WT, *gad2-1*, *gad2-1/GC1::GAD2Δ* #1 and #10 plants, n = 6. **g-h**, Transpiration (**g**), photosynthetic rate (**h**) of 5-6 week-old Arabidopsis WT, *gad2-1* and *gad2-1/GC1::GAD2Δ* #10 plants in response to dark (shaded region) and 150 μmol m^{-2} s^{-1} light (white region), measured using a LiCor LI-6400XT, n = 8 for WT, n = 9 for gad2-1 and n = 8 for *gad2-1/GC1::GAD2Δ* #10 (**g-h**). **i,** WUE of of 5-6 week-old Arabidopsis WT, *gad2-1* and *gad2-1/GC1::GAD2Δ* #10 plants was calculated based on the photosynthetic rate (**h**) against transpiration rate (**g**). All data are plotted with box and whiskers plots: whiskers plot represents minimum and maximum values, and box plot represents second quartile, median and third quartile (**d-f, i**) or data are represented mean ± s.e.m (**g, h**); statistical difference was determined by One-way ANOVA (**d-f, i**), *P <0.05 and ****P <0.0001.

a

WT
(22.2%)

GC1::GAD2Δ #2
(41.7%)

GC1::GAD2Δ #5
(78.6%)

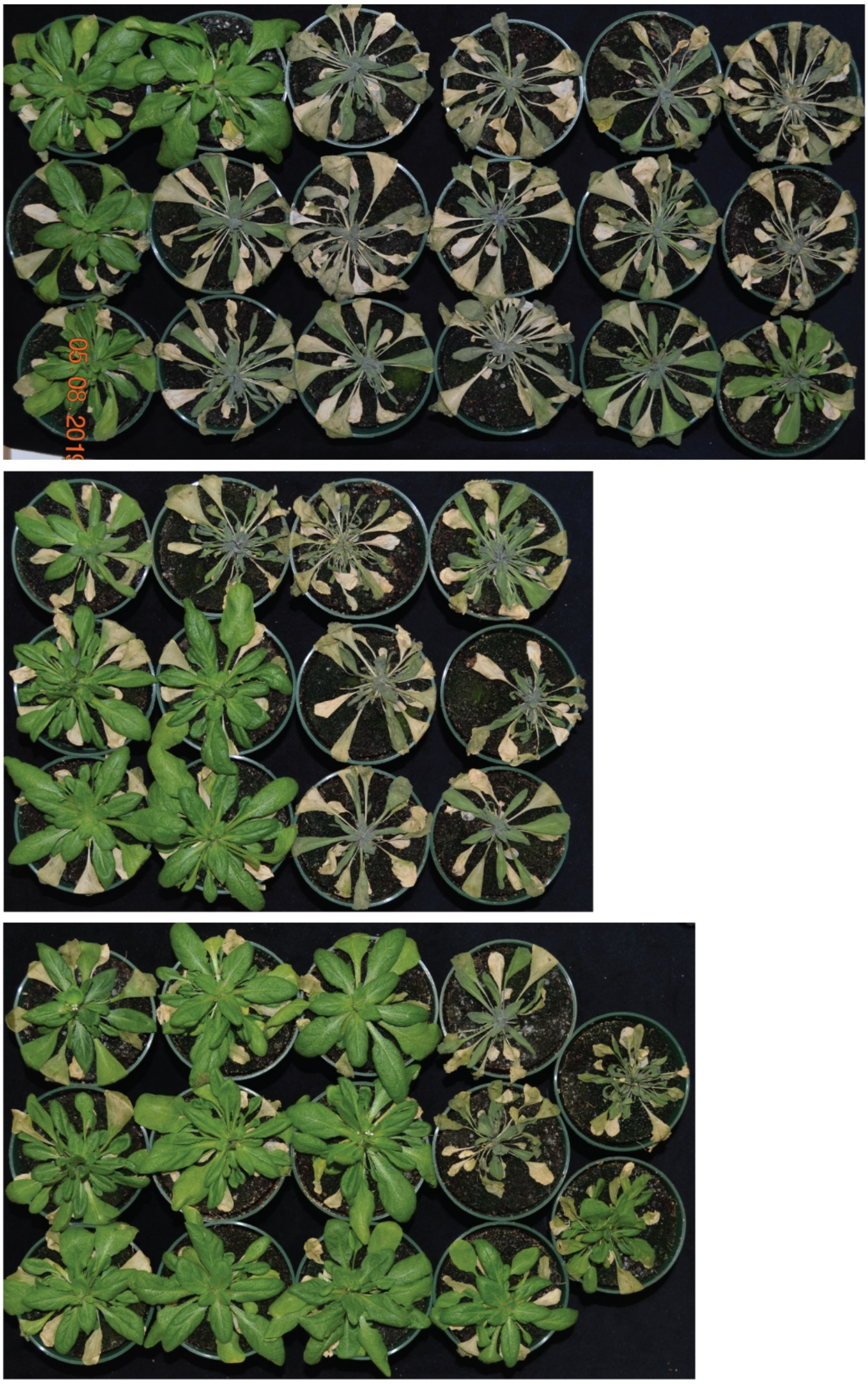

b

WT
(77.8%)

GC1::GAD2Δ #2
(88.9%)

GC1::GAD2Δ #5
(88.9%)

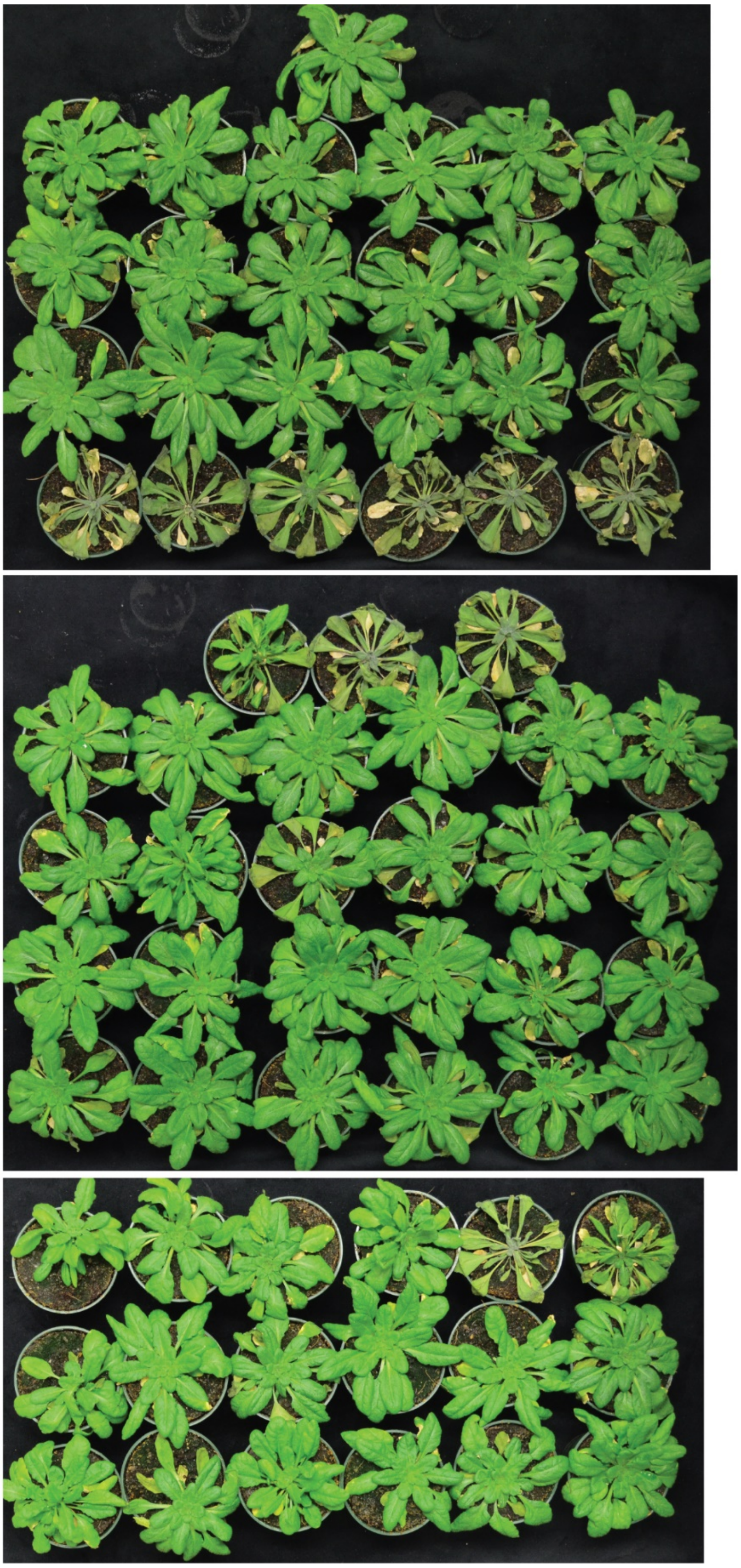

c

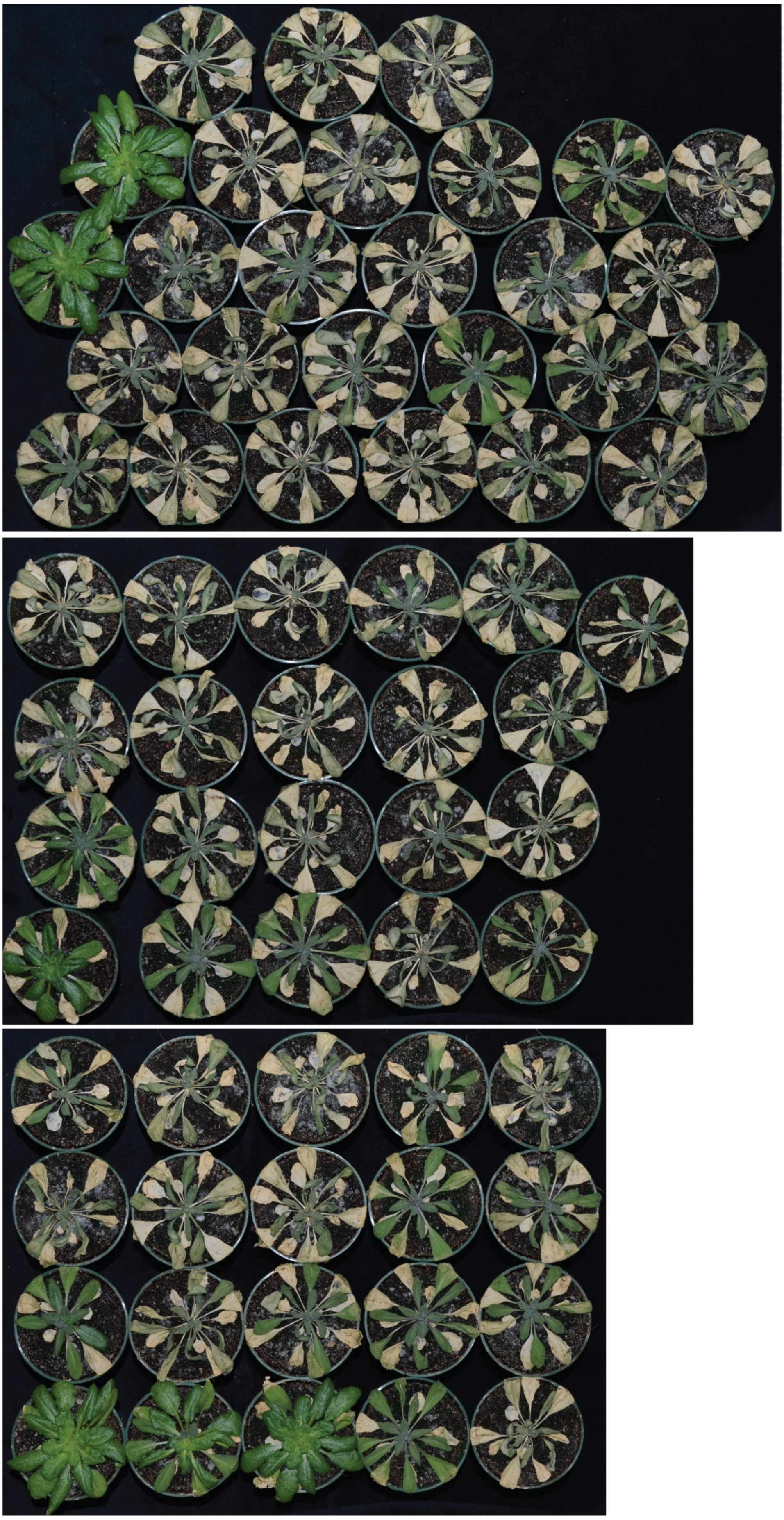

WT
(11.1%)

GC1::GAD2Δ #2
(19.0%)

GC1::GAD2Δ #5
(35%)

Supplementary Figure 9. Recovery of re-watered Arabidopsis WT, *GC1::GAD2Δ* #2 and #5 from drought treatment. a-c, Re-water recovery of wildtype, *GC1::GAD2Δ* #2 and #5 plants from drought in three different batches of experiments, plants were re-watered at 2 days after all plant wilting by drought. A higher recovery rate of *GC1::GAD2Δ* #2 and #5 plants than WT was observed from re-watering in all three experiments (**a-c**); 4 out of 18 (22%) wildtype, 5 out of 12 (41.7%) *GC1::GAD2Δ* #2 and 11 out of 14 (78.6%) *GC1::GAD2Δ* #5 plants were recovered from re-water in the first trail (**a**); 21 out of 27 (77.8%) wildtype, 24 out of 27 (88.9%) *GC1::GAD2Δ* #2 and 16 out of 18 (88.9%) *GC1::GAD2Δ* #5 plants were recovered from re-water in the second trail (**b**); 3 out of 27 (11.1%) wildtype, 4 out of 21 (19.0%) *GC1::GAD2Δ* #2 and 7 out of 20 (35%) *GC1::GAD2Δ* #5 plants were recovered from re-water in the third trail (**c**).

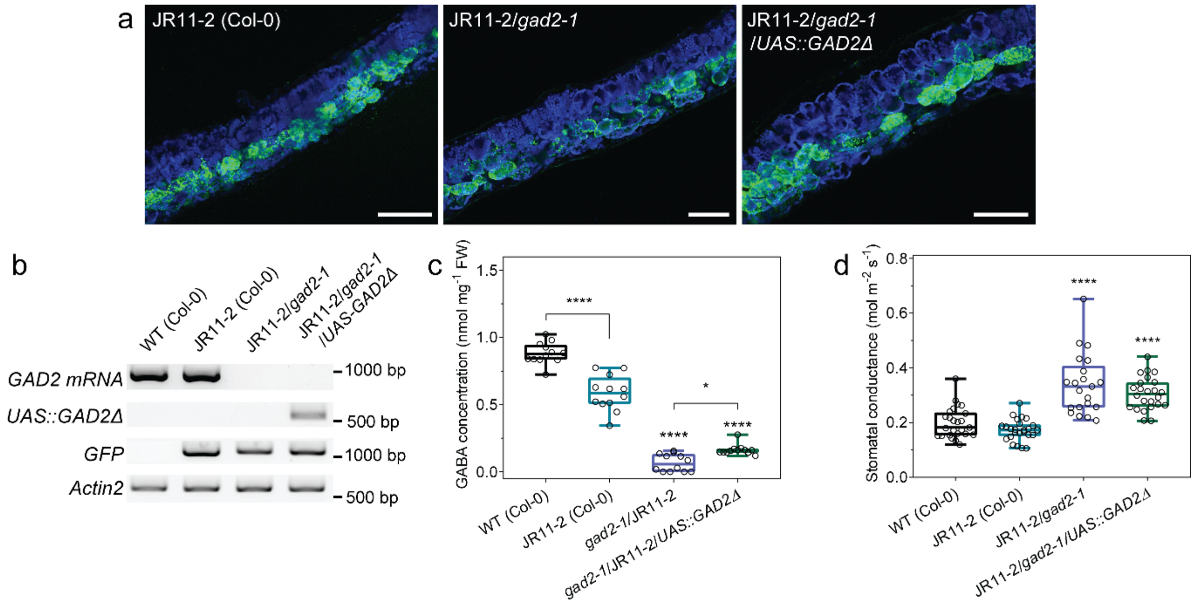

Supplementary Figure 10. Spongy mesophyll-cell specific expression of *GAD2Δ* in *gad2-1* fails to restore stomatal conductance back to wildtype (WT) levels. a, Representative images of leaf transverse cross-sections (30 µm thickness) of 3-4 week-old segregated mesophyll-specific enhancer-trap line JR11-2[5] backcrossed into Arabidopsis Col-0 background[6], JR11-2 in *gad2-1* background (JR11-2/*gad2-1*) and JR11-2/*gad2-1* expressing *UAS::GAD2Δ* (JR11-2/*gad2-1*/*UAS::GAD2Δ*), similar images were obtained from all examined lines, scale bars = 100 µm. GFP fluorescence shown in green indicates cells in which *GAD2* expression will be activated by the yeast transcription factor GAL4, blue indicates chlorophyll autofluorescence. **b,** Reverse-transcriptional PCR quantification of native *GAD2* mRNA (*GAD2mRNA*), *GAD2Δ* driven by *UAS* element (*UAS::GAD2Δ*), *GFP* and *Actin2* transcripts in Arabidopsis WT (Col-0), JR11-2 (Col-0), JR11-2/*gad2-1* and JR11-2/*gad2-1*/*UAS::GAD2Δ* plants, *Actin2* used as an internal control; similar results were obtained from three independent biological replicates. **c-d,** Leaf GABA concentration (**c**) and stomatal conductance (**d**) of 5-6 week-old Arabidopsis WT (Col-0), JR11-2 (Col-0), JR11-2/*gad2-1* and JR11-2/*gad2-1*/*UAS::GAD2Δ* plants, stomatal conductance was measured by AP4 Leaf Porometer (**d**); n = 12 (**c**); n = 25 for WT and JR11-2, n = 21

for JR11-2*/gad2-1* and n = 24 for JR11-2*/gad2-1*/*UAS::GAD2Δ*, data collected from two different batches of plants (**d**). All data are plotted with box and whiskers plots: whiskers plot represents minimum and maximum values, and box plot represents second quartile, median and third quartile (**c, d**); statistically differences were determined by One-way ANOVA by comparing with JR11-2 (**c, d**), $^{*}P < 0.05$ and $^{****}P < 0.0001$.

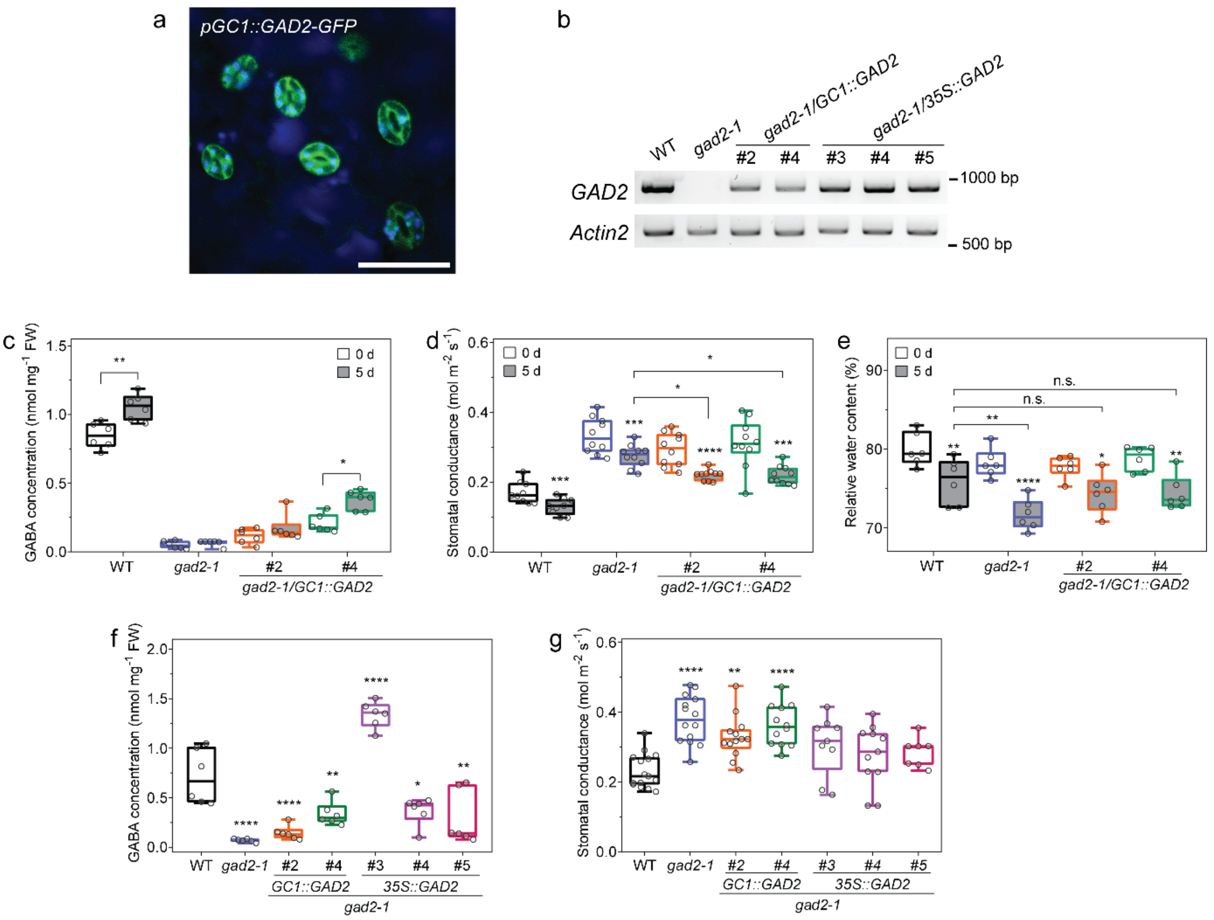

Supplementary Figure 11. Guard-cell specific expression of full-length *GAD2* only reduces the stomatal conductance of *gad2* knockout plants under drought. **a,** Representative confocal image of *gad2*-1 leaves expressing full-length *GAD2* tagged with *GFP* driven by *GC1* promoter (*GC1::GAD2-GFP*), similar images were obtained from all *gad2-1/ GC1::GAD2-GFP* plants examined, scale bar = 50 μm*.* **b,** Reverse-transcriptional PCR quantification of *GAD2* transcripts in wildtype (WT), *gad2-1* and *gad2-1* complementation with full-length *GAD2* driven by guard-cell promoter *GC1* (*gad2-1/GC1::GAD2* #2 and #4) or by a pro35S-CAMV constitutive promoter (*gad2-1/35S::GAD2* #3, #4 and #5), *Actin2* used as an internal control; similar results were obtained from three independent biological replicates. **c-e,** Leaf GABA concentration, stomatal conductance and relative water content of Arabidopsis WT, *gad2-1*, *gad2-1/GC1::GAD2* #2 and #4 plants; n = 6 plants for GABA measurement before (0 d) and after drought treatment for 5 days (5 d) as indicated

(**c**); the stomatal conductance of 5-6 week-old plants was determined by AP4 Leaf Porometer at 0 d and 5 d after drought treatment, n = 9 for WT and n = 10 for *gad2-1*, *gad2-1/GC1::GAD2* #2 and #4 (**d**); relative leaf water content of corresponding leaf samples at 0 d and 5 d after drought treatment, as shown in (**e**). **f-g,** Leaf GABA concentration and stomatal conductance of WT, *gad2-1*, *gad2-1/GC1::GAD2* #2, #4, *gad2-1/35S::GAD2* #3, #4 and #5 plants; n = 6 plants (**f**); the stomatal conductance of WT, *gad2-1*, and *gad2-1* complementation lines; stomatal conductance of 5-6 week-old plants was determined by AP4 Leaf Porometer in normal conditions, n = 15 plants for WT, n = 14 for *gad2-1*, n = 13 for *gad2-1/GC1::GAD2* #2, n = 12 for *gad2-1/GC1::GAD2* #4, n = 9 for *gad2-1/35S::GAD2* #3, n = 11 for *gad2-1/35S::GAD2* #4 and n = 7 for *gad2-1/35S::GAD2* #5 (**g**). All data are plotted with box and whiskers plots: whiskers plot represents minimum and maximum values, and box plot represents second quartile, median and third quartile (**c-g**); statistically differences were determined by One-way ANOVA by comparing with WT (**f, g**), or within genotypes or treatment by Two-way ANOVA (**c-e**), $^{*}P < 0.05$, $^{**}P < 0.01$, $^{***}P < 0.001$ and $^{****}P < 0.0001$.

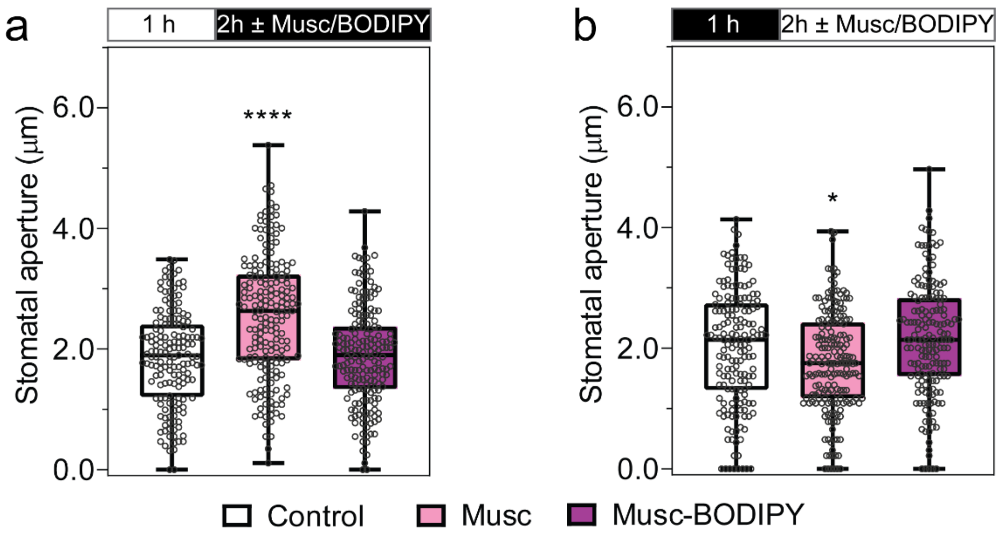

Supplementary Figure 12. Membrane impermeable muscimol does not antagonises stomatal movement initiated by light and dark treatments a-b, Exogenous muscimol-BODIPY application does not affect stomatal movement. Epidermal strips were pre-incubated in stomatal measurement buffer for 1 h under light (**a**) or dark (**b**), followed by 2 h incubation under light-to-dark transition (**a**) or dark-to-light transition (**b**) as indicated above graphs by black (dark) or white (light) bars, together with the application of 10 μM muscimol (Musc) or muscimol-BODIPY (Musc-BODIPY); n = 161 for control, n = 185 for muscimol and n = 188 for muscimol-BODIPY (**c**); n = 168 for control, n = 190 for muscimol and n = 175 for muscimol-BODIPY. All data are plotted with box and whiskers plots: whiskers plot represents minimum and maximum values, and box plot represents second quartile, median and third quartile (**a-b**); statistical difference was determined by One-way ANOVA, **P*<0.05 and *****P*<0.0001.

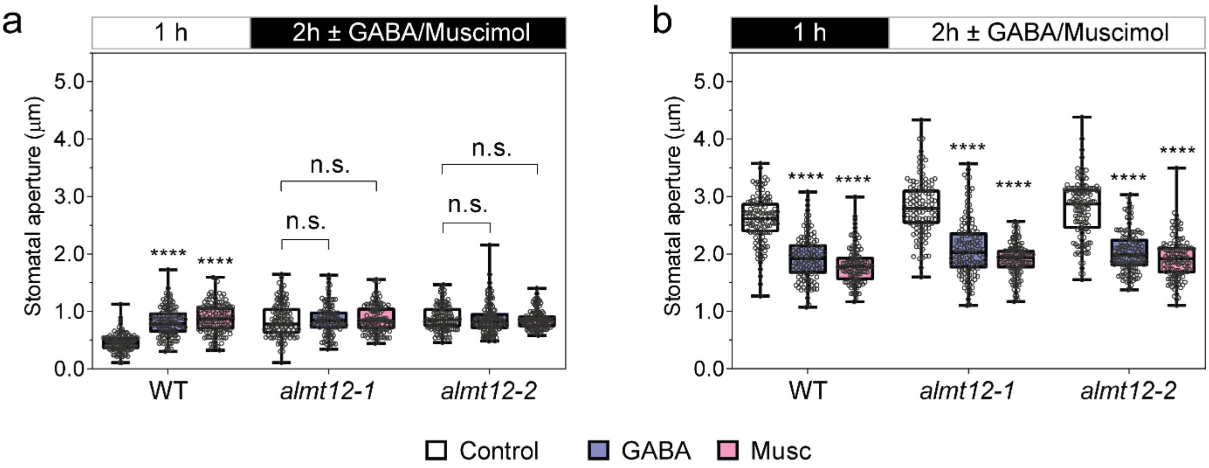

Supplementary Figure 13. Stomatal aperture measurement of WT, *almt12-1* and *almt12-2* knockout plants in response to dark or light. Epidermal strips were pre-incubated in stomatal measurement buffer for 1 h in the light (**a**) or dark (**b**), followed by 2 h incubation in the dark (**a**) or light (**b**) as indicated by black (dark) or white (light) bars above the plots ± 2 mM GABA or 10 μM muscimol (Musc); n = 105 for WT (control), n = 115 for *almt12-1* (control), n = 122 for *almt12-2* (control), n = 122 for WT (GABA), n = 100 for *almt12-1* (GABA), n = 131 for *almt12-2* (GABA), n = 122 for WT (Musc), n = 107 for *almt12-1* (Musc) and n = 118 for *almt12-2* (Musc) (**a**); n = 116 for WT (control), n = 119 for *almt12-1* (control), n = 120 for *almt12-2* (control), n = 113 for WT (GABA), n = 123 for *almt12-1* (GABA), n = 124 for *almt12-2* (GABA), n = 117 for WT (Musc), n = 122 for *almt12-1* (Musc) and n = 116 for *almt12-2* (Musc) (**b**). All data are plotted with box and whiskers plots: whiskers plot represents minimum and maximum values, and box plot represents second quartile, median and third quartile; statistical difference was determined using Two-way ANOVA, ****$P < 0.0001$; all experiments were repeated at least twice from different batches of plants with blind treatments.

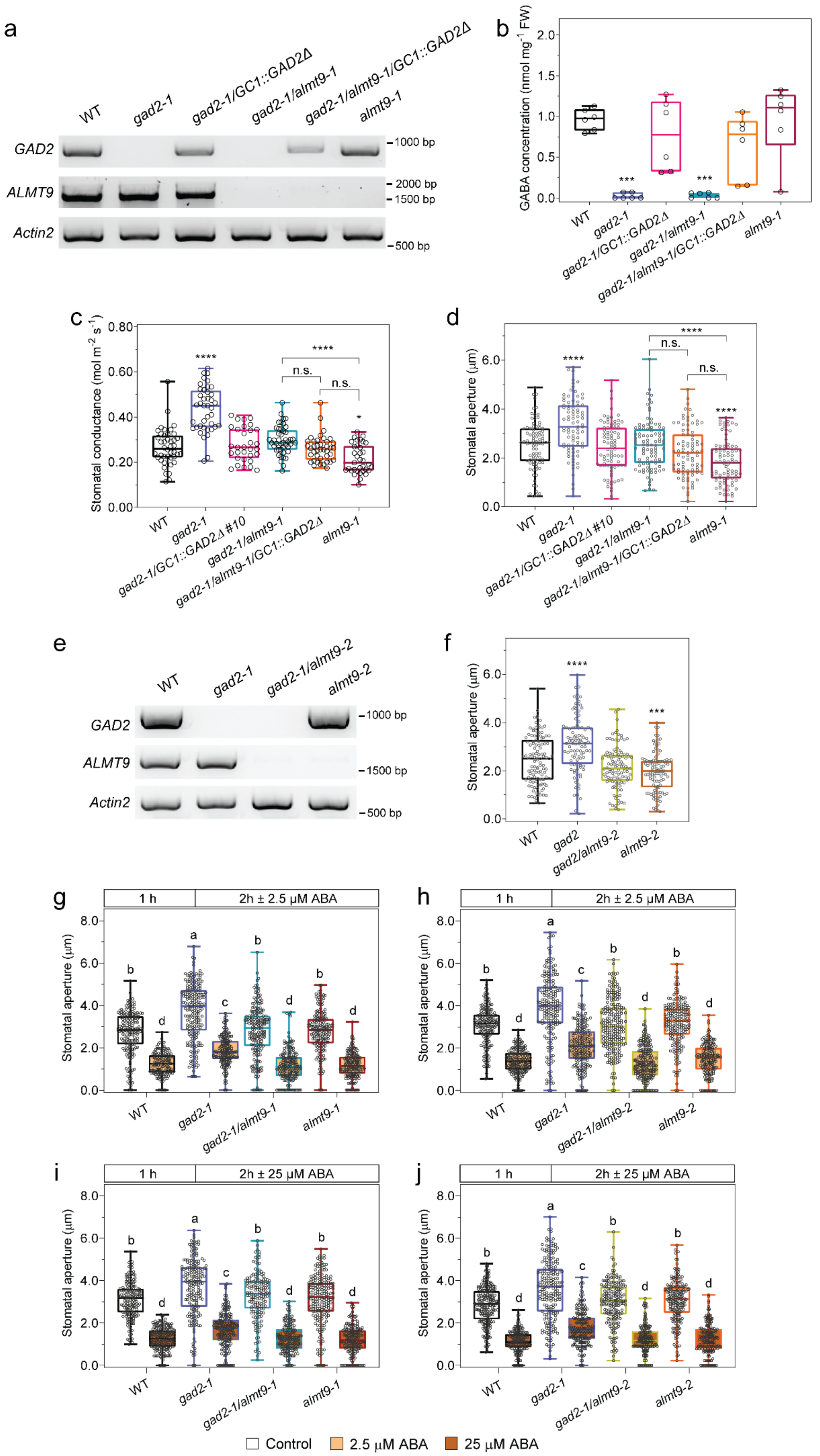
a
WT
gad2-1
gad2-1/GC1::GAD2Δ
gad2-1/almt9-1
gad2-1/almt9-1/GC1::GAD2Δ
almt9-1
GAD2
ALMT9
Actin2
1000 bp
2000 bp
1500 bp
500 bp
b
GABA concentration (nmol mg-1 FW)
***
c
Stomatal conductance (mol m-2 s-1)
gad2-1/GC1::GAD2Δ #10
****
n.s.
*
d
Stomatal aperture (µm)
e
gad2-1/almt9-2
almt9-2
f
gad2
gad2/almt9-2
g
1 h
2h ± 2.5 µM ABA
h
i
2h ± 25 µM ABA
j
Control
2.5 µM ABA
25 µM ABA

Supplementary Figure 14. **The loss of *ALMT9* rescues the larger stomatal aperture and ABA response of *gad2* knockout plants. a,** Reverse-transcriptional PCR quantification of *GAD2*, *ALMT9* and *Actin2* transcripts in Arabidopsis wildtype (WT), *gad2-1*, *gad2-1/GC1::GAD2Δ* #10, *gad2-1/almt9-1*, *gad2-1/almt9-1/GC1::GAD2Δ* and *almt9-1* plants, *Actin2* used as an internal control; similar results were obtained from three independent biological replicates. **b-d,** Leaf GABA accumulation, stomatal conductance and aperture of WT, *gad2-1*, *gad2-1/GC1::GAD2Δ* #10, *gad2-1/almt9-1*, *gad2-1/almt9-1/GC1::GAD2Δ* and *almt9-1* plants*.* The whole rosette leaves of 5-6 week-old plants were harvested for GABA measurement (**b**) and used for stomatal conductance measurement, as determined by AP4 porometer; n = 6 plants (**b**); n = 42 for WT, n = 40 for *gad2-1*, n = 31 for *gad2-1/GC1::GAD2Δ* #10, n = 45 for *gad2-1/almt9-1*, n = 40 for *gad2-1/almt9-1/GC1::GAD2Δ* and n = 35 for *almt9-1*, data collected from four independent batches of plants (**c**). Epidermal strips were peeled and incubated in stomatal measurement buffer for 2 h under light before measurement; n = 86 for WT, *gad2-1*, *gad2-1/GC1::GAD2Δ* #10 and *almt9-1*, n = 95 for *gad2-1/almt9-1* and n = 87 for *gad2-1/almt9-1/GC1::GAD2Δ* (**d**). **e,** Reverse-transcriptional PCR quantification of *GAD2*, *ALMT9* and *Actin2* transcripts in WT, *gad2-1*, *gad2-1/almt9-2* and *almt9-2* plants, *Actin2* used as an internal control; similar results were obtained from three independent biological replicates. **f,** Stomatal aperture of WT (n = 115), *gad2-1* (n = 100), *gad2-1/almt9-2* (n = 106) and *almt9-2* (n = 104) plants; epidermal strips were incubated under light for 2 h before measurement. **g-j**, Stomatal response to ABA of Arabidopsis wildtype (WT), *gad2-1*, *gad2-1/almt9-1*, *almt9-1*, *gad2-1/almt9-2* and *almt9-2* plants. Epidermal strips were pre-incubated in stomatal measurement buffer for 1 h under light, followed by 2 h treatment under light with or without 2.5 μM or 25 μM ABA as indicated; n = 189 (control) and n = 145 (ABA) for WT, n = 208 (control)

and n = 192 (ABA) for *gad2-1*, n = 200 (control) and n = 183 (ABA) for *gad2-1/almt9-1*, n = 182 (control) and n = 181 (ABA) for *almt9-1* (**g**); n = 184 (control) and n = 186 (GABA) for WT, n = 188 (control) and n = 207 (ABA) for *gad2-1*, n = 222 (control) and n = 224 (ABA) for *gad2-1/almt9-2*, n = 197 (control) and n = 182 (ABA) for *almt9-2* (**h**); n = 172 (control) and n = 196 (ABA) for WT, n = 190 (control) and n = 182 (ABA) for *gad2-1*, n = 162 (control) and n = 183 (ABA) for *gad2-1/almt9-1*, n = 192 (control) and n = 181 (ABA) for *almt9-1* (**i**); n = 215 (control) and n =178 (ABA) for WT, n = 197 (control) and n = 174 (ABA) for *gad2-1*, n = 189 (control) and n = 174 (ABA) for *gad2-1/almt9-2*, n = 195 (control) and n =180 (ABA) for *almt9-2* (**j**). All data are plotted with box and whiskers plots: whiskers plot represents minimum and maximum values, and box plot represents second quartile, median and third quartile (**b-d, f, g-i**); statistically differences were determined by One-way ANOVA, *$P < 0.05$, ***$P < 0.001$ and ****$P < 0.0001$ (**b-d, f**), or by Two-way ANOVA, a, b, c and d represent data groups that are not statistically different, $P < 0.05$ (**g-i**).

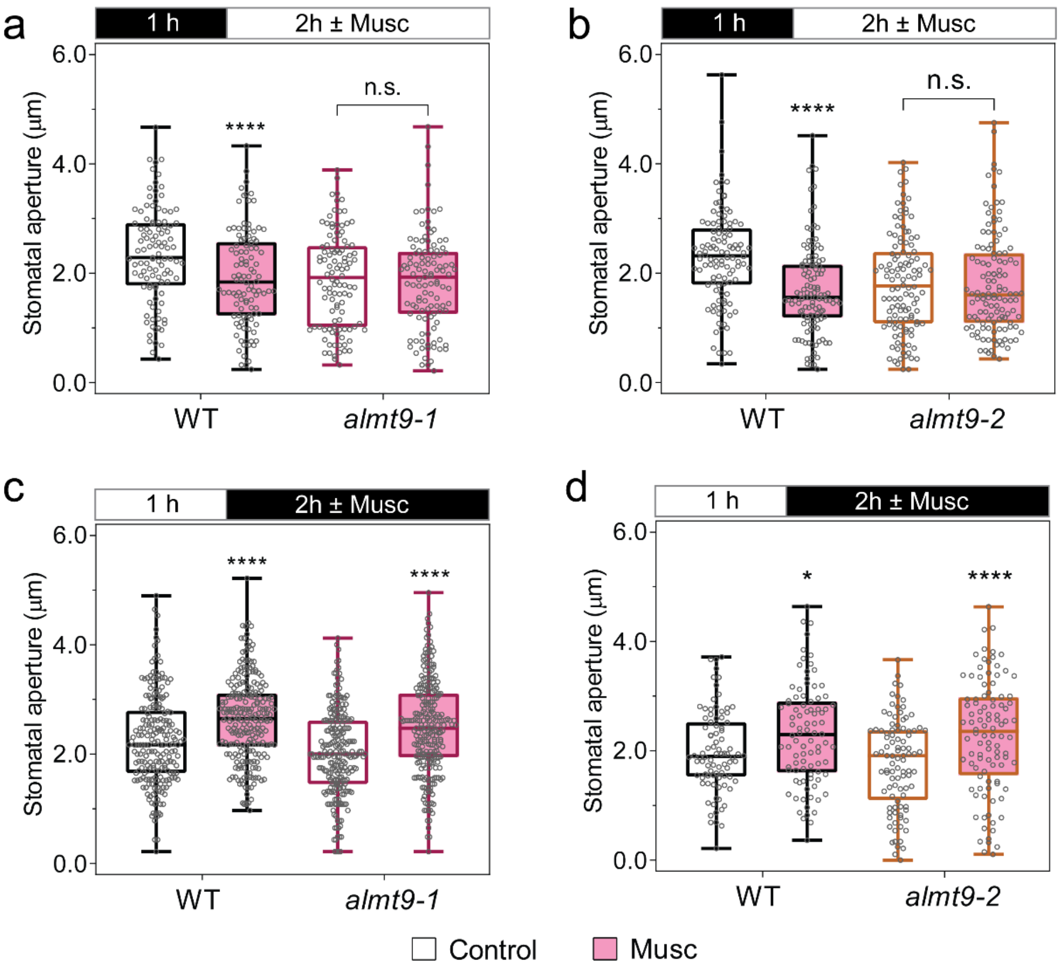

Supplementary Figure 15. *almt9* knockouts abolish muscimol-inhibition of stomatal opening but not affect closure. a-d, Stomatal aperture of wildtype (WT) and *almt9* knockout plants in response to dark or light. Epidermal strips were pre-incubated in stomatal measurement buffer for 1 h under dark (**a-b**) or light (**c-d**), followed by light (**a-b**) or dark (**c-d**) for 2 h as indicated by black (dark) or white (light) bars above graphs, ± 10 µM muscimol (Musc); n = 105 for WT and n = 106 for *almt9-1* with control treatment, n = 106 for WT and n = 111 for *almt9-1* with muscimol treatment (**a**); n = 88 for wildtype (WT) (control); n = 108 for WT (control), n = 116 for *almt9-2* (control), n = 119 for WT (muscimol) and n = 121 for *almt9-2* (muscimol) (**b**); n = 210 for WT and n = 233 for *almt9-1* with control treatment, n = 240 for WT and n = 245 for *almt9-1* with muscimol treatment (**c**); n = 88 for WT (control), n = 96 for *almt9-2* (control), n = 90 for WT (muscimol) and n = 100 for *almt9-2* (muscimol) (**d**); all

experiments were repeated at least twice from different batches of plants with blind treatments (**a-d**). All data are plotted with box and whiskers plots: whiskers plot represents minimum and maximum values, and box plot represents second quartile, median and third quartile (**a-d**); statistically differences were determined by Two-way ANOVA (**a-d**), *$P < 0.05$ and ****$P< 0.0001$.

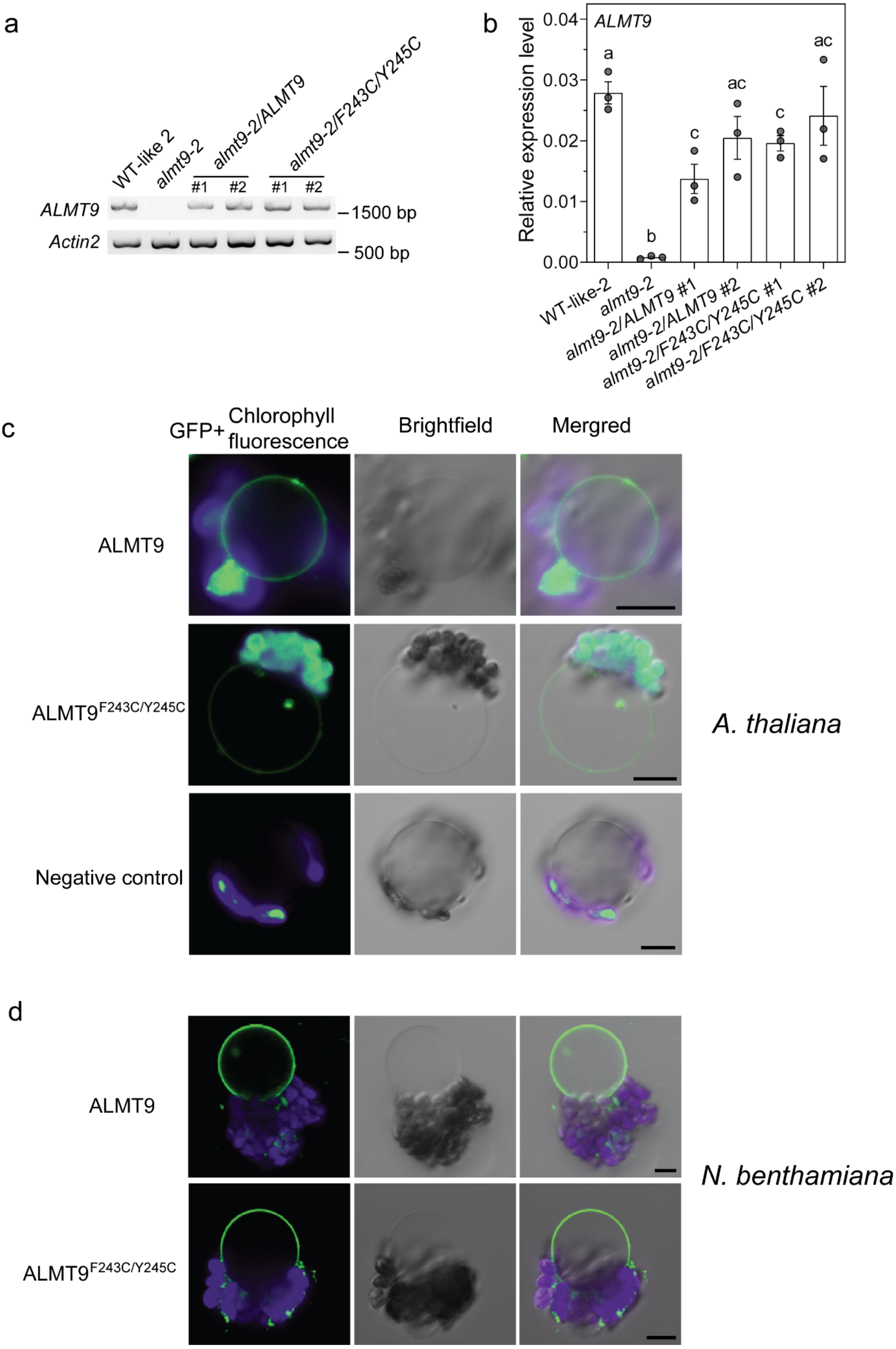

a
WT-like 2
almt9-2
almt9-2/ALMT9
almt9-2/F243C/Y245C
#1
#2
#1
#2
ALMT9
Actin2
1500 bp
500 bp
b
ALMT9
Relative expression level
0.04
0.03
0.02
0.01
0.00
a
b
c
ac
c
ac
WT-like-2
almt9-2
almt9-2/ALMT9 #1
almt9-2/ALMT9 #2
almt9-2/F243C/Y245C #1
almt9-2/F243C/Y245C #2
c
GFP+ Chlorophyll fluorescence
Brightfield
Mergred
ALMT9
ALMT9F243C/Y245C
Negative control
A. thaliana
d
ALMT9
ALMT9F243C/Y245C
N. benthamiana

Supplementary Figure 16. ***ALMT9*** **and** ***ALMT9***$^{\mathbf{F243C/Y245C}}$ **show similar expression in** ***almt9-2*** **complementation lines and both are present on the tonoplast membrane. a-b**, Reverse-transcriptional PCR (**a**) and quantitative real-time PCR (**b**) of *ALMT9* and *Actin2* transcripts in WT-like 2, *almt9-2*, *almt9-2/ALMT9* and *almt9-2/*F243C/Y245C plants. Similar results were obtained from three independent biological replicates (**a**); n = 3 plants and data represented by mean ± s.e.m, statistical difference was determined by two-sided Student's *t*-test, a, b and c represent data groups that are not statistically different, $P < 0.05$ (**b**). **c-d,** Subcellular localisation of ALMT9 and ALMT9$^{F243C/Y245C}$ in Arabidopsis (**c**) and *N. benthamiana* (**d**). Representative confocal image of ALMT9 and ALMT9$^{F243C/Y245C}$ tagged with GFP in the mesophyll protoplasts of *almt9-2/ALMT9* and *almt9-2/*F243C/Y245 complementation lines, repeated on 3 occasions with consistent results (**c**), or transiently expressed in *N. benthamiana* by Agrobacterium infiltration, repeated on 3 occasions with consistent results (**d**); the mesophyll protoplasts of wildtype (WT) Arabidopsis leaves were imaged as control (**c**), using the exact same setting to capture the fluorescence of GFP-tagged ALMT9 and ALMT9$^{F243C/Y245C}$. The mesophyll protoplasts were prepared and lysis as described[7, 8], GFP fluorescence (green) and chlorophyll autofluorescence (purple) captured by Nikon A1R Laser Scanning Confocal (**c, d**).

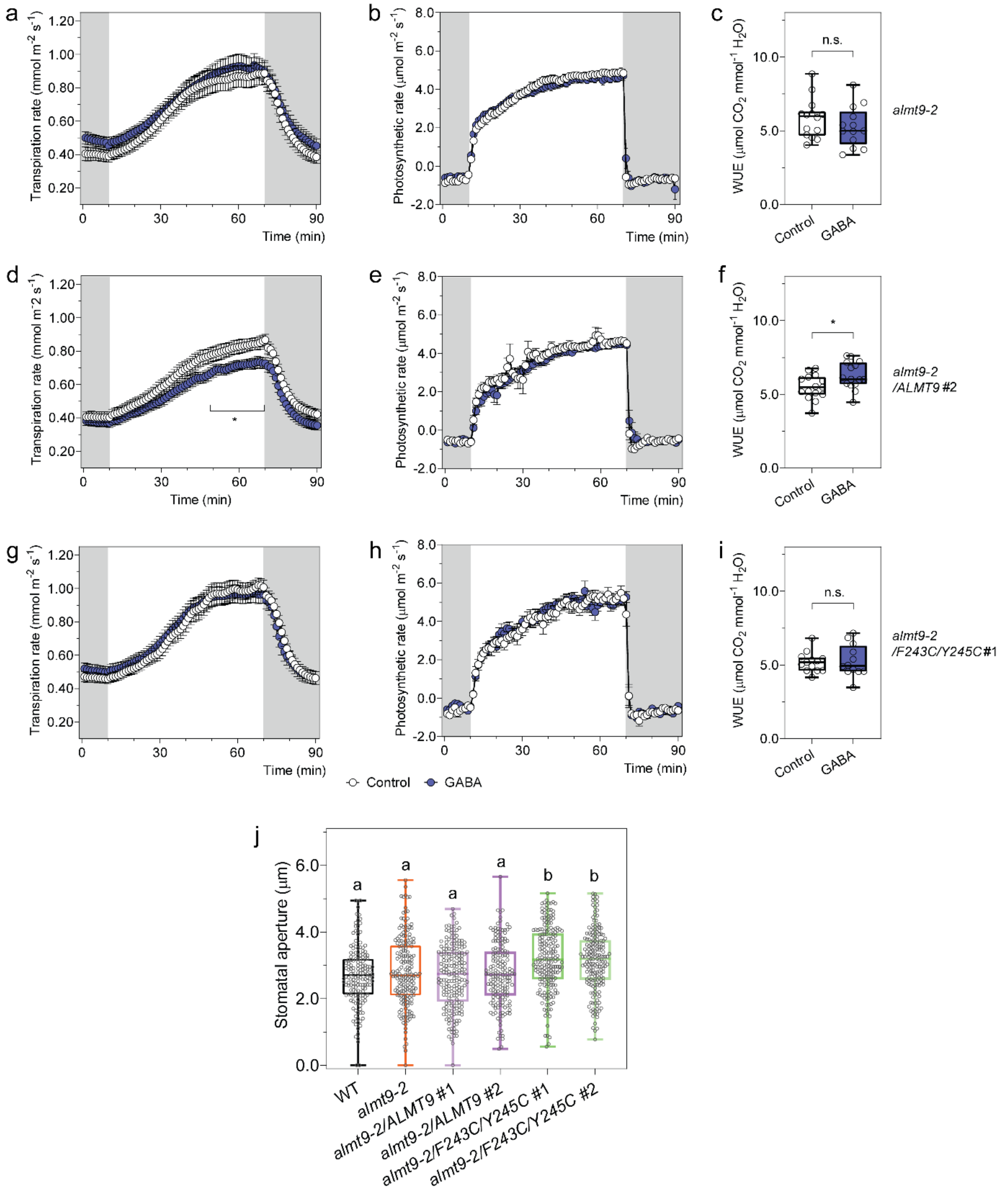

Supplementary Figure 17. *ALMT9* complementation but not by *ALMT9*$^{F234C/245C}$.

a-i, Transpiration, photosynthetic rate and WUE of detached leaves from Arabidopsis *almt9-2* (**a-c**), *almt9-2/ALMT9* #2 (**d-f**) and *almt9-2/*F243C/Y245C #1 (**g-i**) fed with artificial xylem sap solution ± 4 mM GABA using a LiCor LI-6400XT. The WUE of *almt9-2* (**c**), *almt9-2/ALMT9* #2 (**f**) and *almt9-2/*F243C/Y245C #1 (**i**) was calculated the ratio of photosynthetic rate (**b, e, h**) versus transpiration rate (**a, d, g**); n = 14 (control)

and n = 13 (GABA) for *almt9-2* (**a-c**); n = 15 (control and GABA) for *almt9-2/ALMT9* #2 (**d-f**); n = 13 (control) and n = 12 (GABA) for *almt9-2/*F243C/Y245C #1 (**g-i**). **j**, Stomatal aperture of WT, *almt9-2* and complementation lines. Epidermal strips were peeled and incubated in stomatal measurement buffer for 2 h under light before measurement; n = 164 for WT, n = 185 for *almt9-2*, n = 190 for *almt9-2/ALMT9* #1, n = 169 for *almt9-2/ALMT9* #2, n = 198 for *almt9-2/*F243C/Y245C #1 and n = 186 for *almt9-2/*F243C/Y245C #2 (**j**). All data are plotted with box and whiskers plots: whiskers plot represents minimum and maximum values, and box plot represents second quartile, median and third quartile (**c, f, i, j**); or data are represented by means ± s.e.m (**a, b, d, e, g, h**); statistically differences were determined by two-sided Student's *t*-test (**a-i**), *$P < 0.05$; or by One-way ANOVA, a and b represent data groups that are not statistically different, $P < 0.05$ (**j**).

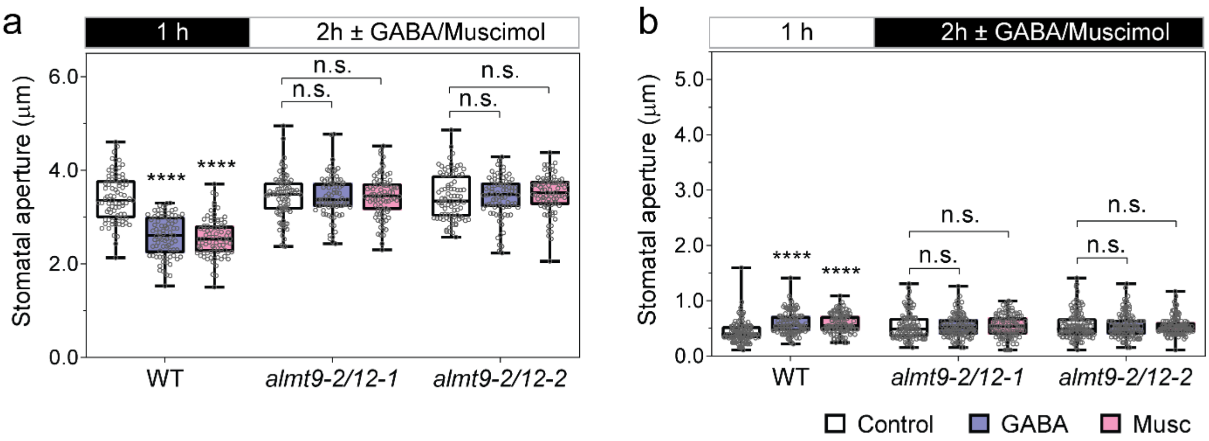

Supplementary Figure 18. The loss of *ALMT9* and *ALMT12* impairs both stomatal opening and closure sensitivity to GABA. Epidermal strips were pre-incubated in stomatal measurement buffer for 1 h in the dark (**a**) or light (**b**), followed by 2 h incubation in the light (**a**) or dark (**b**) as indicated by black (dark) or white (light) bars above the plots ± 2 mM GABA or 10 μM muscimol (Musc); n = 78 for WT (control), n = 82 for *almt9-2/12-1* (control), n = 83 for *almt9-2/12-2* (control), n = 77 for WT (GABA), n = 77 for *almt9-2/12-1* (GABA), n = 81 for *almt9-2/12-2* (GABA), n = 75 for WT (Musc), n = 81 for *almt9-2/12-1* (Musc) and n = 81 for *almt9-2/12-2* (Musc) (**a**); n = 114 for WT (control), n = 104 for *almt9-2/12-1* (control), n = 120 for *almt9-2/12-2* (control), n = 113 for WT (GABA), n = 114 for *almt9-2/12-1* (GABA), n = 123 for *almt9-2/12-2* (GABA), n = 107 for WT (Musc), n = 106 for *almt9-2/12-1* (Musc) and n = 127 for *almt9-2/12-2* (Musc) (**b**). All data are plotted with box and whiskers plots: whiskers plot represents minimum and maximum values, and box plot represents second quartile, median and third quartile; statistical difference was determined using Two-way ANOVA, ****$P < 0.0001$; all experiments were repeated at least twice from different batches of plants with blind treatments.

References

1. Desikan R, Cheung MK, Bright J, Henson D, Hancock JT, Neill SJ. ABA, hydrogen peroxide and nitric oxide signalling in stomatal guard cells. *J Exp Bot* **55**, 205-212 (2004).

2. Melotto M, Underwood W, Koczan J, Nomura K, He SY. Plant stomata function in innate immunity against bacterial invasion. *Cell* **126**, 969-980 (2006).

3. Conn SJ*, et al.* Protocol: optimising hydroponic growth systems for nutritional and physiological analysis of *Arabidopsis thaliana* and other plants. *Plant Methods* **9**, 4 (2013).

4. Sánchez JP, Duque P, Chua NH. ABA activates ADPR cyclase and cADPR induces a subset of ABA-responsive genes in Arabidopsis. *Plant J* **38**, 381-395 (2004).

5. Martí MC, Stancombe MA, Webb A. Cell-and stimulus-type-specific intracellular-free Ca^{2+} signals in *Arabidopsis thaliana*. *Plant Physiol*, **163,** 625-634 (2013).

6. Svozil J, Gruissem W, Baerenfaller K. Proteasome targeting of proteins in Arabidopsis leaf mesophyll, epidermal and vascular tissues. *Front Plant Sci* **6**, 376 (2015).

7. De Angeli A, Zhang J, Meyer S, Martinoia E. AtALMT9 is a malate-activated vacuolar chloride channel required for stomatal opening in Arabidopsis. *Nat Commun* **4**, 1804 (2013).

8. Yoo S-D, Cho Y-H, Sheen J. Arabidopsis mesophyll protoplasts: a versatile cell system for transient gene expression analysis. *Nat Protoc* **2**, 1565 (2007).
