# Supplementary material for: GABA signalling modulates stomatal opening to enhance plant water use efficiency and drought resilience
Source: Nat Commun. 2021 Mar 29;12:1952. doi: 10.1038/s41467-021-21694-3 (PMC8007581; doi:10.1038/s41467-021-21694-3)
Supplement: Supplementary file 3 — Reporting Summary [file 41467_2021_21694_MOESM3_ESM.pdf]

nature research

Corresponding author(s): Matthew Gilliham

Last updated by author(s): Feb 2, 2021

Reporting Summary

Nature Research wishes to improve the reproducibility of the work that we publish. This form provides structure for consistency and transparency in reporting. For further information on Nature Research policies, see Authors & Referees and the Editorial Policy Checklist .

Statistics

For all statistical analyses, confirm that the following items are present in the figure legend, table legend, main text, or Methods section.

n/a	Confirmed	
☐	☒	The exact sample size (*n*) for each experimental group/condition, given as a discrete number and unit of measurement
☐	☒	A statement on whether measurements were taken from distinct samples or whether the same sample was measured repeatedly
☐	☒	The statistical test(s) used AND whether they are one- or two-sided<br>*Only common tests should be described solely by name; describe more complex techniques in the Methods section.*
☐	☒	A description of all covariates tested
☒	☐	A description of any assumptions or corrections, such as tests of normality and adjustment for multiple comparisons
☐	☒	A full description of the statistical parameters including central tendency (e.g. means) or other basic estimates (e.g. regression coefficient) AND variation (e.g. standard deviation) or associated estimates of uncertainty (e.g. confidence intervals)
☐	☒	For null hypothesis testing, the test statistic (e.g. *F*, *t*, *r*) with confidence intervals, effect sizes, degrees of freedom and *P* value noted<br>*Give P values as exact values whenever suitable.*
☒	☐	For Bayesian analysis, information on the choice of priors and Markov chain Monte Carlo settings
☒	☐	For hierarchical and complex designs, identification of the appropriate level for tests and full reporting of outcomes
☒	☐	Estimates of effect sizes (e.g. Cohen's *d*, Pearson's *r*), indicating how they were calculated

Our web collection on statistics for biologists contains articles on many of the points above.

Software and code

Policy information about availability of computer code

Data collection	N/A
Data analysis	N/A

For manuscripts utilizing custom algorithms or software that are central to the research but not yet described in published literature, software must be made available to editors/reviewers. We strongly encourage code deposition in a community repository (e.g. GitHub). See the Nature Research guidelines for submitting code & software for further information.

Data

Policy information about availability of data

All manuscripts must include a data availability statement. This statement should provide the following information, where applicable:

- Accession codes, unique identifiers, or web links for publicly available datasets
- A list of figures that have associated raw data
- A description of any restrictions on data availability

All raw data is available for figures in manuscript, no data availability restrictions apply, all accession codes have been included.

Field-specific reporting

Please select the one below that is the best fit for your research. If you are not sure, read the appropriate sections before making your selection.

☒ Life sciences ☐ Behavioural & social sciences ☐ Ecological, evolutionary & environmental sciences

For a reference copy of the document with all sections, see nature.com/documents/nr-reporting-summary-flat.pdf

Life sciences study design

All studies must disclose on these points even when the disclosure is negative.

Sample size	We calculated based on power analysis of the variation within our stomatal aperture measurements that 60 stomata were sufficient to gain significance between treatments, we took no less than 100 measurements per genotype per treatment. All plant experiments are based on at least 6 replicates per genotype per treatment which follows accepted convention for normally distributed data in such experiments. Only Fig. 4g and Suppl. Fig 7, 16b have less than 6 replicates due to the large number of genotype comparisons. However the data is consistent with 3 other repeats of drought sensitivity of the complemented plants.
Data exclusions	No data points have been excluded from the analyses.
Replication	All experiments were repeated at least twice and some four times.
Randomization	All plants were arranged randomly and rearranged daily to avoid local growth environments impinging upon results. Samples sent for GABA measurements were randomised and analyzed by Empower chromatography software version 3 (Waters). Measurements of treatments for apertures and conductance were randomised.
Blinding	All treatments in stomatal aperture experiments and GABA measurement were conducted blind.

Reporting for specific materials, systems and methods

We require information from authors about some types of materials, experimental systems and methods used in many studies. Here, indicate whether each material, system or method listed is relevant to your study. If you are not sure if a list item applies to your research, read the appropriate section before selecting a response.

Materials & experimental systems

n/a	Involved in the study
☒	☐ Antibodies
☒	☐ Eukaryotic cell lines
☒	☐ Palaeontology
☒	☐ Animals and other organisms
☒	☐ Human research participants
☒	☐ Clinical data

Methods

n/a	Involved in the study
☒	☐ ChIP-seq
☒	☐ Flow cytometry
☒	☐ MRI-based neuroimaging
